# Factorized binary search: Change point detection in the network structure of multivariate high-dimensional time series

**DOI:** 10.1162/imag_a_00520

**Published:** 2025-04-17

**Authors:** Martin Ondrus, Emily Olds, Ivor Cribben

**Affiliations:** Neuroscience and Mental Health Institute, University of Alberta, Alberta, Canada; Alberta School of Business, University of Alberta, Alberta, Canada

**Keywords:** change point detection, time series, high-dimensional, NMF, fMRI, network analysis

## Abstract

Functional magnetic resonance imaging (fMRI) time series data present a unique opportunity to understand the behavior of temporal brain connectivity, and models that uncover the complex dynamic workings of this organ are of keen interest in neuroscience. We are motivated to develop accurate change point detection and network estimation techniques for high-dimensional whole-brain fMRI data. To this end, we introduce*factorized binary search*(FaBiSearch), a novel change point detection method in the network structure of multivariate high-dimensional time series in order to understand the large-scale characterizations and dynamics of the brain. FaBiSearch employs non-negative matrix factorization, an unsupervised dimension reduction technique, and a new binary search algorithm to identify multiple change points. In addition, we propose a new method for network estimation for data between change points. We seek to understand the dynamic mechanism of the brain, particularly for two fMRI data sets. The first is a resting-state fMRI experiment, where subjects are scanned over three visits. The second is a task-based fMRI experiment, where subjects read Chapter 9 of*Harry Potter and the Sorcerer’s Stone*. For the resting-state data set, we examine the test–retest behavior of dynamic functional connectivity, while for the task-based data set, we explore network dynamics during the reading and whether change points across subjects coincide with key plot twists in the story. Further, we identify hub nodes in the brain network and examine their dynamic behavior. Finally, we make all the methods discussed available in the R package fabisearch onCRAN, as well as all experiments onGitHub.

## 1 Introduction

Functional magnetic resonance imaging (fMRI) is widely used as an indirect method of measuring brain activity using the blood-oxygen-level-dependent (BOLD) signal (Ogawa et al., 1990). With increases in neuronal activity, glucose and oxygen must be delivered to the neurons from the blood stream, causing a local increase in oxyhemoglobin. This subsequently increases the ratio of oxyhemoglobin to deoxyhemoglobin, and provides the basis of measurement of the BOLD signal in fMRI. Typically, multiple slices of the subject’s brain are scanned and then divided into small (a few millimeters in dimension) cubes called voxels. By taking multiple scans of each slice sequentially over time, a time series of voxel activity can be created.

With the advent of the ever increasing computational capacity, and general interest in scalable, data-driven techniques for characterizing neurological phenomena, fMRI data present a unique opportunity. By studying the relationships among voxels (or a cluster of voxels often referred to as regions of interest, or ROIs), we can better characterize brain connectivity leading to a greater understanding of the brain and extensive clinical implications (Bullmore & Sporns, 2009;Sporns, 2010). Functional connectivity (FC) seeks to define the relationships (Biswal et al., 1995) by means of correlation, covariance, and precision matrices among other techniques (seeCribben & Fiecas, 2016for a review). Graph theory remains at the core of most FC-based estimation methods, and for good reason; it intuitively represents the brain as a graphical model with ROIs as nodes and temporal dependence as edges. These FC relationships have vast scientific and clinical implications. Not only can FC be used to understand brain processes during tasks or resting, but also to characterize clinical diagnoses. For example, schizophrenia has long been associated with abnormal FC, characterized by disorder in network patterns such as small-worldness (Nejad et al., 2012).

Many FC methods assume that the connections between brain regions remain static throughout the experiment, simplifying analysis by reducing computational complexity. However, this assumption neglects the dynamic nature of neural processes, where brain connectivity fluctuates in response to cognitive changes. Evidence from both resting-state data (Delamillieure et al., 2010;Doucet et al., 2012) and task-based experiments (Debener et al., 2006;Eichele et al., 2008;Fox et al., 2005) reveals that FC is non-stationary, reflecting evolving patterns of synchronization over time. Traditional models that treat brain circuitry as fixed are unable to capture these fluctuations, limiting their ability to account for responses to task progression—such as the unfolding of a narrative during a reading task. AsVarela et al. (2001)suggest, neural assemblages continuously adjust their synchronization in response to internal and external stimuli. Therefore, understanding the dynamic aspects of FC requires examining not only which brain regions synchronize, but also how this synchronization evolves over time and under different stimuli (Gonzalez-Castillo & Bandettini, 2018).

Numerous statistical methods have emerged to capture and express the dynamic nature of FC. First, sliding window approaches were introduced that extend covariance, correlation, or precision matrix methods into a time-varying context. These approaches define sequential blocks of time points and estimate FC within each block. By estimating FC across blocks from the beginning to the end of the time course, time-varying FC can be estimated (seeHutchison et al., 2013for a review). Although the sliding window approach is a computationally practical way of determining FC, it has limitations (Hutchison et al., 2013). For one, the resulting FC patterns are heavily influenced by the choice of block size, which can result in vastly different FC patterns. Additionally, this technique gives no weight to time points outside of those included in the window.

Instead of a moving window, change point methods have also been applied to this problem. Here, the objective is to find the optimal windows for stationary structures. There exists an extensive literature and a long history on change points beginning withPage (1963). The most widely discussed problems have been concerned with finding change points in univariate time series. and more recently, with detecting multiple change points in multivariate time series. For example,Aue et al. (2009)introduced a method to detect changes in the covariance matrix of a multivariate time series,Dette and Wied (2016)proposed a test where the dimension of the data is fixed, while more recentlyKao et al. (2018)considered the case where the dimension of the data increases with the sample size (they also investigated change point analysis based on principal component analysis).Sundararajan and Pourahmadi (2018)proposed a new method for detecting multiple change points in the covariance structure of a multivariate piece-wise stationary process. Using a combination of principal components analysis and wavelets to transform the time series,Cho and Fryzlewicz (2015)segmented the multivariate time series into partitions based on the second-order structure.

There have also been many new change point methods developed specifically for an application to neuroimaging data.Cribben et al. (2012)first introduced a change point method for estimating dynamic functional connectivity by considering change points in precision matrices (undirected graphs) using binary segmentation and the Bayesian Information Criterion metric. Accordingly,Cribben et al. (2013);Schröder and Ombao (2019);Kirch et al. (2015);Gibberd and Nelson (2014);Avanesov and Buzun (2018);Dai et al. (2019);Anastasiou et al. (2022)then proposed further methods for estimating FC change points.Koepcke et al. (2016);Mosqueiro et al. (2016);Xiao et al. (2019)also considered change points in spike trains. While these methods are effective, they all are limited in the number of time series that can be considered. Subsequently, there has been a drive to extend techniques to high-dimensional spaces, specifically the casep>>T, wherepis the number of time series andTis the total length of the time series. To this end,Cribben and Yu (2017)introduced the network change point detection method, which uses both change point and community detection techniques to estimate change points by examining the time evolving community network structure of multivariate time series. In addition,Ofori-Boateng et al. (2021)introduced a new method that first presents each network snapshot of fMRI data as a linear object and finds its respective univariate characterization via local and global network topological summaries and then adopts a change point detection method for (weakly) dependent time series based on efficient scores. While these methods are an adequate starting point to understand dynamic neural processes, the true granularity in which the brain functions is lost with less comprehensive cortical maps of the brain. In practice, these cortical maps define fewer ROIs for the same size of cerebrum, hence activity is averaged across a larger surface area. This decreases the specificity of individual ROI activity and effectively assumes that activity is the same within each, which is highly unlikely given the complexity of most cognitive processes.

More recently,Candemir et al. (2021)presented an fMRI-based method for detecting emotional changes during social-support tasks, using a three-phase approach to account for noise and variability in brain signals.Kim et al. (2021)proposed a method for detecting change points in dynamic functional connectivity using the maximum eigenvalues of covariance matrices within a random matrix theory framework, with applications to epilepsy data demonstrating its ability to identify meaningful changes in brain activity.Li and Li (2023)suggest an online change point detection method for identifying shifts in the covariance structure of high-dimensional data, with a stopping rule which accommodates spatial and temporal dependencies without requiring Gaussian assumptions, and a theoretical framework for determining detection thresholds efficiently.Baek et al. (2023)discuss three methods (dynamic connectivity regression, max-type, and PCA-based) for detecting change points in correlation networks to identify significant shifts in dynamic processes, such as brain state transitions in fMRI data.

There has also been recent research to improve binary segmentation.Fryzlewicz (2014)proposed wild binary segmentation, which uses random localization to estimate multiple change points accurately.Eichinger and Kirch (2018)examined the statistical properties of change point estimators using moving sum statistics, extending previous results by accommodating random exogenous change points, and proving consistent estimation of both the number and locations of change points.Kovács et al. (2023)introduced seeded binary segmentation for scalable change point detection, using deterministic background intervals to improve reproducibility and computational speed, achieving near-linear runtimes.Cho and Fryzlewicz (2024)presented WCM.gSa, which combines wild contrast maximization to handle serial correlations with a gappy Schwarz algorithm for consistent estimation of change points and dependence structure.Verzelen et al. (2023)proposed optimal detection and localization rates for change points in time series, revealing a phase transition from global detection to local estimation, and introduced two efficient procedures that achieve these rates even with low-energy, undetectable change points present. In high-dimensional, large-scale data sets, traditional binary segmentation is computationally inefficient, as each time point must be individually evaluated. More recent methods such as wild and seeded binary segmentation partially address this but rely on random sampling, adding complexity that limits scalability. This motivates the need for a structured, efficient approach to handle the demands of high-dimensional change point detection without added computational burden.

As such, we are motivated to develop accurate change point detection and network estimation techniques for high-dimensional whole-brain fMRI data. We seek to understand the dynamic mechanism of the brain through these techniques, particularly for two experiments using theGordon et al. (2016)ROI atlas (p=333). The first data set is a test–retest resting-state fMRI experiment of 25 participants scanned over 3 visits. Here, we examine the change in an individual’s brain dynamics behavior over time within a scanning session, the test–retest reliability of dynamic functional connectivity (across scans or longitudinal measurements), and whether commonalities exist across subjects’ estimated networks or functional states. The second data set is a task-based fMRI experiment of eight subjects reading a chapter of*Harry Potter and the Sorcerer’s Stone*(Rowling, 2012). Again, using a large number of brain regions (p=333), we seek to understand the behavior of whole-brain network dynamics, but crucially, we hope to understand how the change points and estimated networks coincide with key features in the plot.

To this end, we introduce a new method, called factorized binary search (FaBiSearch), to detect multiple change points in the network (or clustering) structure of multivariate high-dimensional time series. FaBiSearch has the following unique and important attributes. First, FaBiSearch is, to the best of our knowledge, the first statistical method to use non-negative matrix factorization (NMF) for finding change points in the network (or clustering structure) in multivariate high-dimensional time series, which allows us to understand the large-scale behavior and dynamics of the brain. NMF is an unsupervised dimension reduction technique which learns a parts-based representation of a non-negative input data matrix. NMF has many properties that make it suitable and attractive for change point detection in multivariate high-dimensional time series data. For example, it has an inherent clustering (or community) property, where it automatically groups together variables, which is useful in neural data where the activity of brain regions can synchronize based on stimuli (Gonzalez-Castillo & Bandettini, 2018;Varela et al., 2001) in a low-rank manner (Yatsenko et al., 2015). Additionally, compared with other dimension reduction techniques, NMF does not have any constraints on the interaction of variables to be orthogonal, as in principal component analysis (PCA:Pearson, 1901) or independent, as in independent component analysis (ICA:Jutten & Herault, 1991). Such constraints do not necessarily hold for fMRI data or for multivariate time series data in general. Consequently, NMF is more flexible in modeling the interactions between variables and allows for some overlap in the basis components (Lee & Seung, 1999). NMF also scales to high dimensions given its dimension reduction property (Brunet et al., 2004;Devarajan, 2008;Lee & Seung, 1999;Xu et al., 2003), therefore, making it especially relevant to settings wherep>>T. Due to the many desirable properties, NMF has been increasingly used in neuroimaging research as a favored method over more traditional approaches (Anderson et al., 2014;Chen et al., 2023;Lohmann et al., 2007;Won et al., 2022;Xie et al., 2017).

Second, FaBiSearch is suitable for detection of multiple change points, which are common not only in task-based fMRI experiments, but also in resting-state experiments. Third, FaBiSearch is scalable in that it is not limited by the dimensionality of the problem space and is, therefore, ideal for characterizing large, changing network structures, such as those in the brain. For the simulated data sets we consider in this work, we find that FaBiSearch has a superior performance to previous state-of-the-art methods. In addition, for simulations where the subject alternates between two states (such as task-based fMRI data) and where the subject transitions between states, FaBiSearch performs very well and clearly outperforms the other methods. Fourth, for FaBiSearch, we introduce a novel binary search algorithm to identify multiple change points in a high-dimensional setting. This new change point algorithm dramatically increases computational efficiency over binary segmentation. Fifth, the NMF element of FaBiSearch allows us to define a new method for the estimation of networks for data between change points, which provides a visual display of the clustering structure and the FC networks between the brain regions. Sixth, while motivated by fMRI data, FaBiSearch is a general change point detection method, hence FaBiSearch may also be applied to other brain imaging modalities such as electroencephalography (EEG), magnetoencephalography (MEG), and electrocorticography (ECoG), and other multivariate high-dimensional time series data where the network, community, or clustering structure is changing over time.

The setup of this paper is as follows. InSection 2.1we define notation for the method, while inSection 2.2, we describe the problem setting and goal of our approach. InSection 2.3, we describe our novel method, FaBiSearch, and inSection 2.4, we describe the method for estimating networks between pairs of change points. InSection 2.5, we describe the simulated and fMRI data sets and we outline the results inSection 3. We have a discussion inSection 4before concluding inSection 5. The R package**fabisearch**implements these methods, and is available on CRAN (Ondrus & Cribben, 2021). Code for experiments is available on GitHub athttps://github.com/mondrus96/FaBiSearch_exp.

## 2 Methods

### 2.1 Preliminaries

An entry in theith row andjth column of a matrixAis denoted by$A_{ij}$. A matrix is non-negative,A≥0, if and only if all entries of the matrix are non-negative, that is,$A_{ij}\geq 0$∀ i,j. For a matrix of time series data$X\in {\mathbb{R}}^{T\times p}$,Tis the total number of discrete time points andpis the number of variables. The set ofknumber of*true*change points inXis denoted by$Q^{*}=\{q_{1}^{*},\ldots ,q_{k}^{*}\},$where$1<q_{1}^{*}<q_{2}^{*},\ldots ,q_{k}^{*}<T$. For some pair of time points$t_{1},t_{2}<T$where$t_{1}<t_{2}$, we denote the corresponding range of samples inXas$X_{t_{1}:t_{2},}$. Similarly, for some pair of variable indices$p_{1},p_{2}<p$where$p_{1}<p_{2}$, we denote the corresponding range of columns inXas$X_{,p_{1}:p_{2}}$. We denote the cardinality of a set with||. We defineδas the minimum distance between two change points, which is equivalent to the minimum sample size in our setup. We refer to the set of all ordered (from smallest to largest) possible change points asQ={1+δ,…,T−δ},mcandidate change points as$\hat{Q}=\{{\hat{q}}_{1},\ldots ,{\hat{q}}_{m}\}$, andschange points as${\hat{Q}}^{*}=\{{\hat{q}}_{1}^{*},\ldots ,{\hat{q}}_{s}^{*}\}$, where${\hat{Q}}^{*}\subseteq \hat{Q}\subseteq Q\subseteq \left[1,\ldots ,T\right]$.

### 2.2 Problem setting

Consider a high-dimensional, multivariate time series$X=\{x_{t}\in {\mathbb{R}}^{p}:t=1,2,\ldots ,T\},$wherexis a vector ofpvariables at timet, andp>Tor possibly*p*>>*T*. Further, assume that the joint distribution across allpvariables ofXis stationary, with structural breaks defined by$Q^{*}=\{q_{1}^{*},q_{2}^{*},\ldots ,q_{k}^{*}\}$. In the case thatXdoes not have change points,k=0andQis an empty set, andXis stationary. IfXis non-stationary, we segmentXintok+1stationary segments$S_{i}$denoted byQ, such that$S_{i}=\{x_{t}\in {\mathbb{R}}^{p}:q_{i-1}<t\leq q_{i}\}$. Our goal in this setting is that, givenX, we are interested in recoveringQ*without any information on the location or number of true change points.

### 2.3 Change point model setup

In this section, we introduce our Factorized Binary Search (FaBiSearch) method. To do so, we first describe non-negative matrix factorization (NMF), then our novel segmentation method, and finally the permutation test procedure. Our method is an unsupervised, offline (Aminikhanghahi & Cook, 2017), and test-based approach for change point detection. A test-based approach attempts to first find candidate change points through partitioning of the time series, and then evaluate the candidates through permutation testing (Antoch & Hušková, 2001), which has been extensively applied in the literature (Anastasiou & Fryzlewicz, 2022;Andrews, 1993;Chen & Zhang, 2015;Cribben et al., 2012;James et al., 1987;Shao & Zhang, 2010;Xiong & Cribben, 2022).

#### 2.3.1 Non-negative matrix factorization

NMF is a matrix factorization technique which constrains the input matrix and subsequent factors to non-negative values (Lee & Seung, 2000). It is commonly used as an unsupervised dimension reduction method which projects data into simpler factors, making it naturally useful in high dimensions (Brunet et al., 2004;Devarajan, 2008;Lee & Seung, 1999;Xu et al., 2003). It also implicitly clusters the time series, which is important in neuroimaging where it is of interest to find communities (or functional states) that correspond to closely knitted groups of time series (or nodes).

**Fig. 1.**
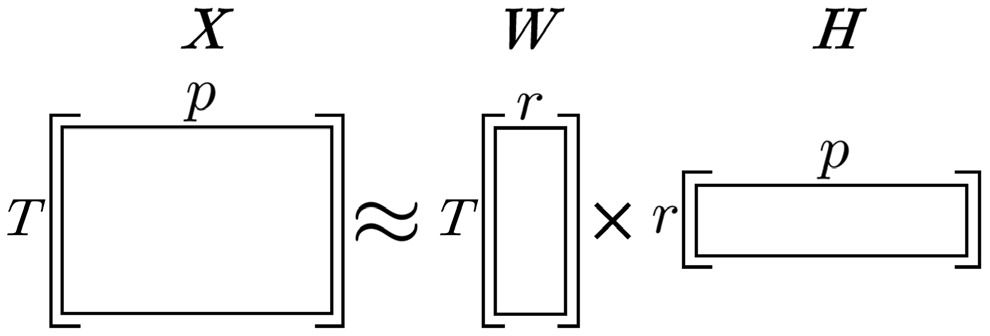
A graphic of non-negative matrix factorization (NMF), where$X\in {\mathbb{R}}^{T\times p}$is approximated by the product of low-dimensional factors$W\in {\mathbb{R}}^{T\times r}$and$H\in {\mathbb{R}}^{r\times p}$, wherer<<min(T,p).

More formally, consider a non-negative matrix ofTsamples andpvariables,$X\geq 0\in {\mathbb{R}}^{T\times p}$. NMF seeks to minimize the distance between the original matrixX, and the product of two low-rank factors, a coefficient matrix,$W\geq 0\in {\mathbb{R}}^{T\times r}$, and a basis matrix,$H\geq 0\in {\mathbb{R}}^{r\times p}$with rankr∈ℕ<<min(T,p)(Fig. 1), such thatX≈WH. The interpretation of rank is context dependent, however, due to the clustering property of NMF (Li & Ma, 2004;Li et al., 2014), it can be seen as the number of unique clusters inX. The value ofris chosen*a priori*and should be large enough thatWHretains the key information ofXwhile also small enough that the addition of noise has a minimal effect; choosingris key for managing the bias-variance trade-off.

We utilize a loss measure based on the generalized Kullback–Leibler divergence fromLee and Seung (2000)(or*I*-divergence:Honkela et al., 2011) to assess model fit,

$$\text{KLD}(X||WH)=\underset{\begin{array}{c} i,j \end{array}}{\sum }\left(X_{ij}\text{log}\frac{X_{ij}}{{\left(WH\right)}_{ij}}-X_{ij}+{\left(WH\right)}_{ij}\right),$$ (1)

where the asymmetric divergence between the input matrixXand the NMF factorizationWHis calculated for each entry in theith row andjth column. In addition to this loss metric,Lee and Seung (2000)also describe other loss metrics and algorithms utilizing multiplicative updates to find factorsWandHgiven an input matrixX. Given NMF is non-convex (Lee & Seung, 2000), NMF is typically run with multiple random initializations, which we denote as$n_{\text{run}}$, to help convergence.

As mentioned previously, rankris a key parameter in NMF, however, in many cases it is unknown. Thus, to find the optimal rank, we provide a data-driven solution for its estimation, which we define as*optrank*, which has been adapted fromFrigyesi and Höglund (2008). Let$X^{\prime }$be the matrix resulting from randomly permuting the columns and rows ofX. Furthermore, let$WH_{r}$and$WH_{r}^{\prime }$be the estimated matrices using NMF onXand$X^{\prime }$, respectively, at some rankr. The approach compares the improvement in loss from increasing rank inXand$X^{\prime }$, and stops when the improvement in loss is no better than noise. More formally, starting atr=1, calculate$\Delta_{X}\leftarrow \text{KLD}(X||WH_{r+1})-\text{KLD}(X||WH_{r})$and$\Delta_{X^{\prime }}\leftarrow \text{KLD}(X^{\prime }||WH_{r+1})-\text{KLD}(X^{\prime }||WH_{r})$. Iterate throughrwhile$\Delta_{X}>\Delta_{X\prime }$, and then the optimal rank,$r^{*}$, is the firstr, where$\Delta_{X}<\Delta_{X\prime }$.

We are, in particular, interested in a change in the model residuals as reflected by the Kullback–Leibler Divergence. A change point at time$t=q^{*}$then is formally defined as a point where there is a statistically significant shift in the mean of${\text{KLD}}_{t}$, which indicates a change in the divergence between the original data and the NMF reconstructed approximation.

#### 2.3.2 Segmentation using binary search

The most commonly used segmentation method for change point detection is binary segmentation (Douglas & Peucker, 1973;Duda & Hart, 1974;Ramer, 1972), where a univariate collection of samples is sequentially evaluated across the time index set. This can then be extended to the multivariate time series setting, by choosing some representative criterion which we sequentially evaluate across the time index set. In particular, for data$X\in {\mathbb{R}}^{T\times p}$, binary segmentation proceeds such that each time pointt∈[1...T]is evaluated sequentially for some chosen model measure,L(e.g., loss, likelihood, or some information criterion). The time point which minimizes or maximizes the measure is picked as the candidate change point,$\hat{q}$, and this process is recursively applied to find multiple change points. This class of methods is broadly referred to as “top down” (Keogh et al., 2004) or “forward selection” (Niu et al., 2016) algorithms. Typical adaptations of this method include the addition of a minimum sample size or distance between change pointsδ, and hypothesis testing to evaluate the candidate change point(s). However, binary segmentation has some issues. First, it is inefficient and equivalent to carrying out a brute-force search of change points which becomes exacerbated when each model fitment itself is expensive. Second, it has been shown that it is not effective for the multiple change point setting (Fryzlewicz, 2014;Niu et al., 2016).

Instead of evaluating all possible time points, we propose a novel efficient method to identify multiple candidate change points which considers a binary search method that has been adapted for change point detection. Binary search, also called half-interval search (Williams Jr, 1976) or binary chop (Butterfield et al., 2016), is a foundational algorithm typically used to find the position of values in a sorted list. In FaBiSearch, we apply binary search at each parent segment, which has a worst-case computational complexity of orderO(logT)forTindices (Cormen et al., 2022), compared with complexity of orderO(TlogT)for binary segmentation (seeFryzlewicz, 2014for a discussion). We provide a description of the binary search method for change point detection inAlgorithm 1.

**Algorithm 1:** Binary search algorithm (binsearch) adapted for change point detection for input time series$X\in {\mathbb{R}}^{T\times p}$. Anymodeland loss metric,L, can be chosen.

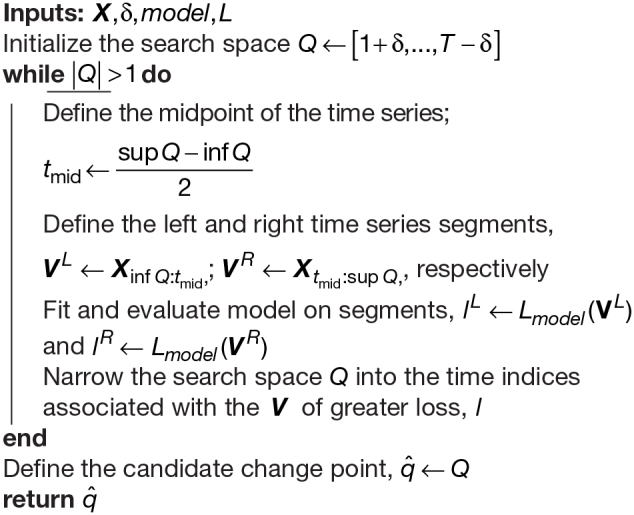

To describe our binary search method, let us first consider a one change point setting. Implicit in binary segmentation is the idea that for some$t\in q^{*}$and some shift away from this (ε),$L_{model}(X_{1:t+\varepsilon ,})+L_{model}(X_{t+\varepsilon +1:T,})>L_{model}(X_{1:t,})$$+L_{model}(X_{t+1:T,})$. More generally, the sum of losses of two segments increases as the distance from the true change point increases. This can be attributed to an inferior model fit and, therefore, higher loss in the segment which contains the candidate change point, which is loosely related to the idea of optimistic search inKovács et al. (2020). This is precisely what underlies the mechanisms ofAlgorithm 1, wherein at each iteration, we consider two segments$V^{L}$and$V^{R}$which overlap at the midpoint ofX, and narrow the search space to the segment with higher loss since it is more likely to contain a candidate change point. Each iteration of this process cuts the search spaceQapproximately in half until the length of the time index equals1, which signals the end of the process. The candidate change point,$\hat{q}$, is simply the remaining time index inQ. This is summarized with an example graphic inFigure 2. Similar to binary segmentation, once$\hat{q}$is detected,Xis then split into two child segments$X_{1:\hat{q}}$and$X_{\hat{q}+1:T}$. Naturally then, the algorithm can be applied recursively to each child segment to find multiple change points, as long asT≥1in the child segments.

**Fig. 2.**
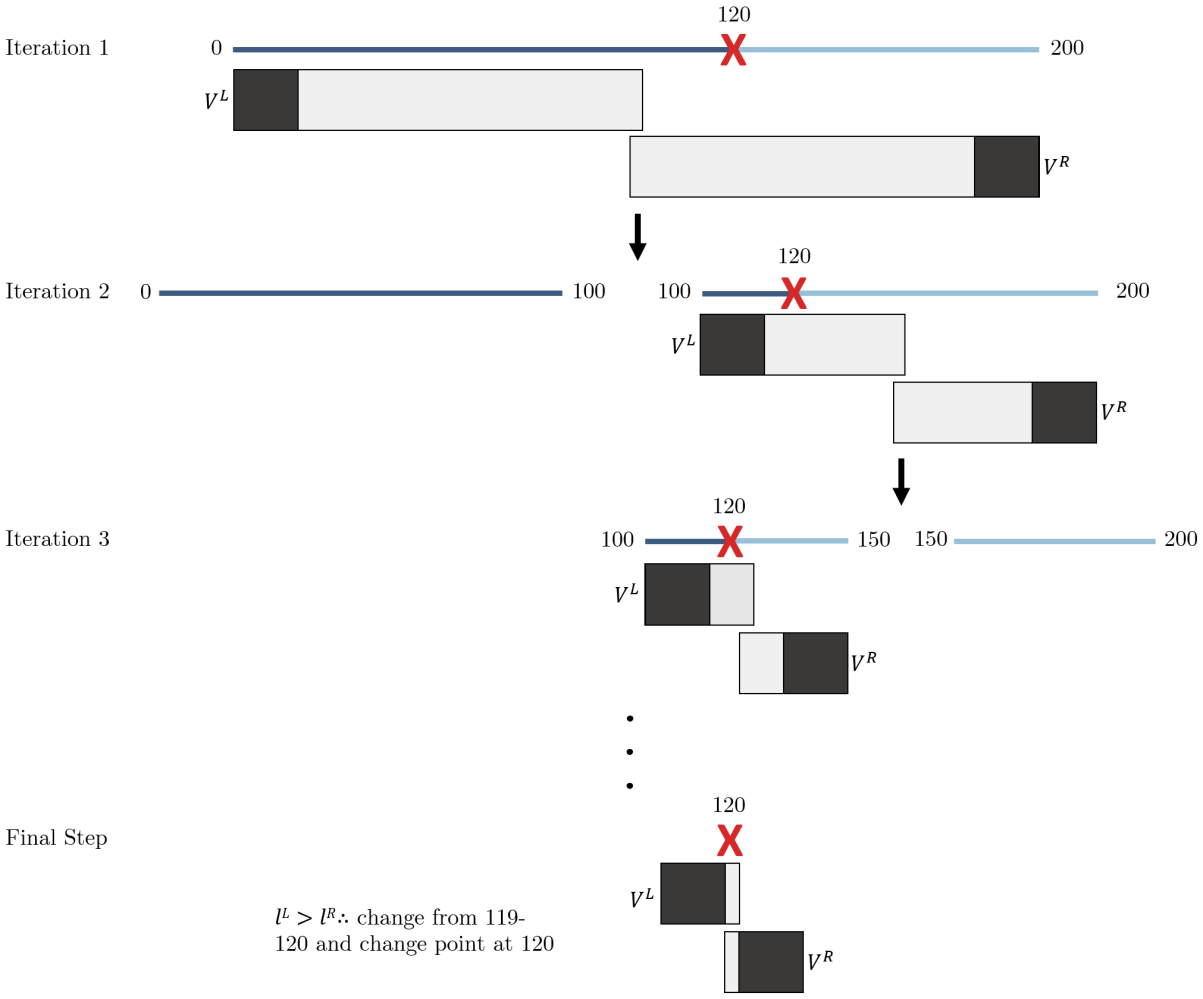
A graphic of the modified binary search procedure for FaBiSearch. The multivariate time series (T=200) denoted by the dark blue and light blue lines is progressively cut in half to find the true change point ($q^{\text{*}}=120$) denoted by the red cross. Below each time series are the two (left and right) blocks of data where NMF is estimated. Each block consists ofδworth of padding at the outermost edges (black), and the overlap of the two blocks (gray) in the middle.

The advantages of the binary search approach are twofold. First, by iteratively halving the search space, binary search improves upon the sequential, exhaustive search of binary segmentation by reducing the number of required fitments of the model. This technique enables more efficient change point detection in larger datasets. Second, by successively narrowing the search space at each iteration, the influence of other change points in a multiple change point context becomes diminished. As a result, this approach also improves upon the accuracy of change point detection. For finite sample performance, we refer readers toAppendix Awhich provides a comparison of binary search to binary segmentation in a multiple change point simulation study.

#### 2.3.3 Refitting segments and statistical inference

Once the binary search algorithm has been exhausted, and we have detected candidate change points$\hat{Q}$, we carry out a refitting procedure. Since our strategy is to overestimate the number of change points by recursively segmentingX, we next prune the number of candidate change points detected using the following method which fits more broadly within the class of permutation tests (Antoch & Hušková, 2001;Fisher, 1936). This two-phase approach is closely related to “tree pruning” in the context of classification and regression trees (Breiman, 2017). For the general case, themcandidate change points arranged in ascending order are denoted by$\hat{Q}=\left\{{\hat{q}}_{1},{\hat{q}}_{2},...,{\hat{q}}_{m}\right\}$. Then, we divideXinto the set ofm+1predicted stationary segments$\hat{S}=\{{\hat{S}}_{1},{\hat{S}}_{2},...,{\hat{S}}_{m+1}\}$, where theith segment is denoted by${\hat{S}}_{i}=X_{b_{i}-1:b_{i},}$where$b=\left\{1,\hat{Q},T\right\}$is the set of candidate change points inclusive of the end points1andT.

For each${\hat{q}}_{i}$, we define the pair${\hat{S}}_{i}$and${\hat{S}}_{i+1}$which are the stationary segments immediately before and after theith candidate change point. Next, we define${\hat{S}}_{i}^{\prime }$and${\hat{S}}_{i+1}^{\prime }$which are obtained from permuting${\hat{\text{S}}}_{\;i}^{\prime }\cup {\hat{\text{S}}}_{i+1}^{\prime }$over time points, and splitting at${\hat{q}}_{i}$. This step effectively disrupts any existing temporal ordering and provides a reference of no change point structure. The loss at${\hat{q}}_{i}$is$L_{model}\left({\hat{\text{S}}}_{i}\right)+L_{model}\left({\hat{\text{S}}}_{i+1}\right)$, while the permuted loss is$L_{model}\left({\hat{S}}_{i}^{'}\right)+L_{model}\left({\hat{S}}_{i+1}^{'}\right)$. We repeat this procedure for$n_{reps}$number of repetitions, generating distributions over the$n_{reps}$samples of the unpermuted and permuted loss,$l_{i}$and$l_{i}^{\prime }$, respectively.

To determine whether a candidate change point is a change point, we are interested in the following hypothesis:

$$\begin{array}{c} H_{0\;\;}:\;\;\mu_{l_{i}}\geq \mu_{l_{i}^{\prime }}\\ H_{a}\;\;:\;\mu_{l_{i}}<\mu_{l_{i}^{\prime }}. \end{array}$$

We use Welch’st-test and construct the test statistic as follows:

$$t=\frac{{\bar{l}}_{i}-l_{i}^{\prime }}{\sqrt{\frac{s_{i}^{2}}{N_{i}}+\frac{s_{i}^{\prime 2}}{N_{i}^{\prime }}}}.$$

In the case we have multiple candidate change points, we adjust thep-value in each statistical test (for each candidate change point) for multiple comparisons using the method fromBenjamini and Hochberg (1995). Each candidate change point in$\hat{q}$becomes a change point${\hat{q}}^{*}$if and only if we reject$H_{0}$for a givenα. This process is repeated for each candidate change point in$\hat{Q}$. The complete FaBiSearch method using NMF is described inAlgorithm 2.

**Algorithm 2:** FaBiSearch algorithm for multiple change point detection.

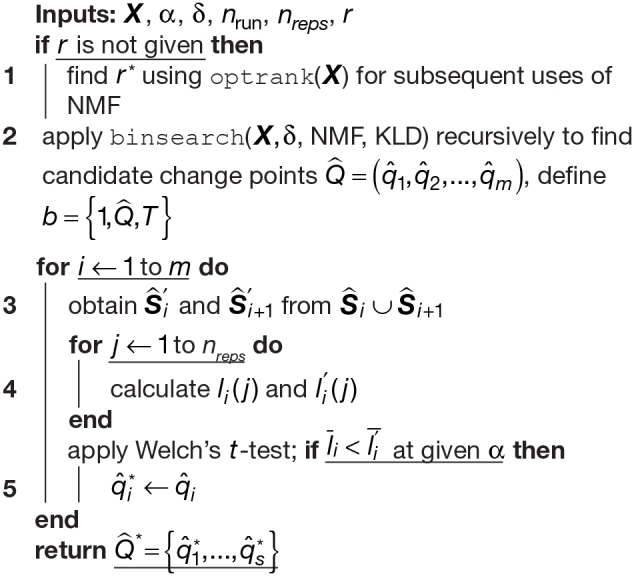

### 2.4 Estimating stationary networks

We introduce an NMF-based method to compute an adjacency matrix using clustering, which is based on previous work fromBrunet et al. (2004). For each stationary block of data,${\text{S}}_{i}$, we first apply NMF. Cluster membership is then determined from ther×pcoefficient matrix,H. Specifically, each column ofH(representing a time series) is assigned to the cluster corresponding to the row with the highest coefficient value. For each run in$n_{\text{runs}}$, an adjacency matrix,

$${\text{A}}_{ij}=\{\begin{array}{cc} 1, & \text{if}\;i,j\;\text{are in the same cluster};\\ 0, & \text{otherwise} \end{array}$$

is generated representing the cluster membership of each node (which represents each time series). By taking the average of all adjacency matrices generated over$n_{\text{runs}}$, we obtain the corresponding consensus matrix,

$$\text{C}=\frac{\sum_{k=1}^{n_{\text{runs}}}({\text{A}}^{k})}{n_{\text{runs}}},\;\;0\leq {\text{C}}_{ij}\leq 1\;\;\forall i,j\text{.}$$

This procedure has the advantage that it can combine results across$n_{\text{run}}$, which may be unfavorable on their own, into an overall matrix where each entry denotes the probability of two nodes being clustered together. This is similar to stability selection inMeinshausen and Bühlmann (2010)and bootstrapping inZhu and Cribben (2018). From here, we apply a clustering algorithm (such as hierarchical clustering with complete linkage) to classify cluster membership among nodes in the consensus matrix. This provides a clustering tree which we then cut at a predetermined number of clusters which, in practice, we compute by using the optimal rank from the change point detection step. The final product is an adjacency matrix computed as an ensemble of multiple NMF runs. The new method is shown inFigure 3. While this method provides an intuitive way to organize nodes into clusters, it requires a prespecified number of communities to be defined. In some cases or applications, this may not be known and it may not be a convenient way of interpreting interactions among nodes. Further, there is no way to adjust for a level of sparsity and thus many networks may be too dense to easily interpret. As such, we also define relationships among nodes using the consensus matrix values and using a prespecified threshold,λ, to control the sparsity/density of the resulting adjacency matrix:

**Fig. 3.**
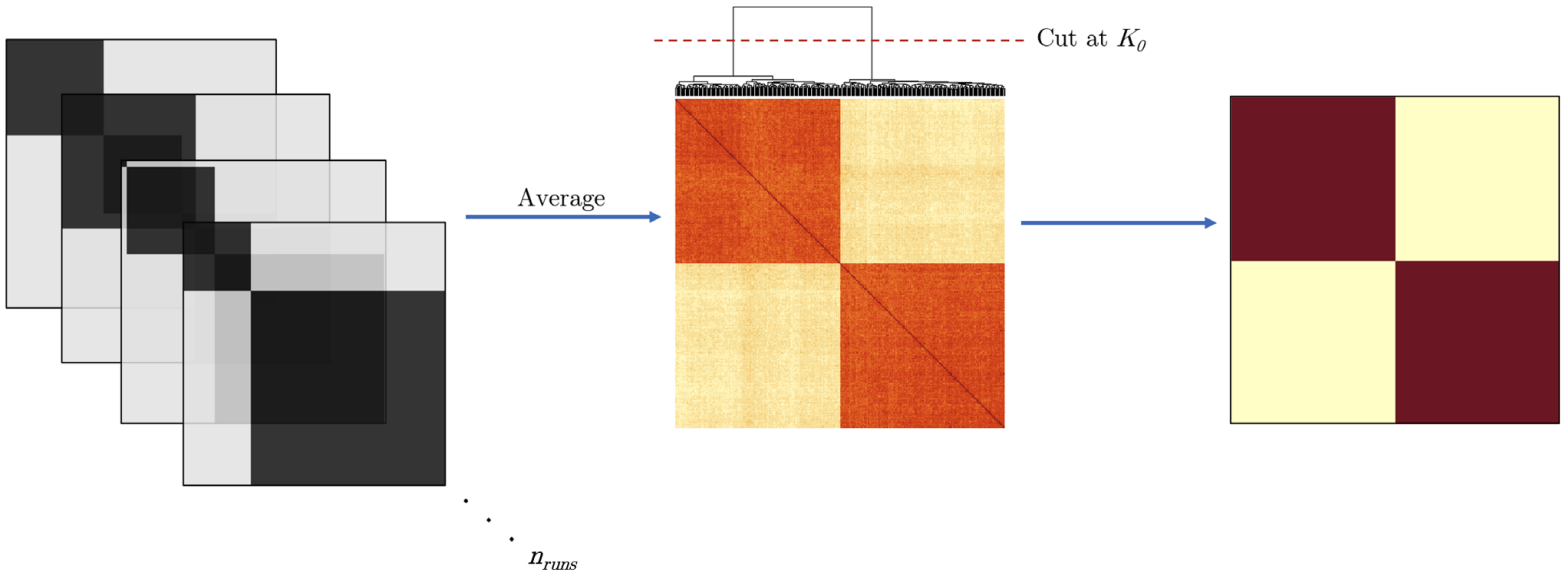
The workflow for estimating networks between pairs of change points (stationary blocks). Individual adjacency matrices are calculated over$n_{\text{runs}}$and then averaged to find the consensus matrix. Using a clustering algorithm, the consensus matrix is determined. Finally, the tree is cut at a prespecified$K_{0}$value and the nodes are then assigned cluster membership.

$${\text{A}}_{ij}=\{\begin{array}{cc} 1, & \text{if}\;{\text{C}}_{ij}>\lambda ;\\ 0, & \text{otherwise}. \end{array}$$

### 2.5 Data

#### 2.5.1 Simulation study setup

We apply our FaBiSearch method to multiple simulations across various dimensions. As a measure of accuracy of the detected locations in time, we compare the detected change points with the location of the true change points using the scaled Hausdorff distance,

$$d_{H}=n_{s}^{-1}\;\text{max}\left\{\underset{j}{\text{max}}\underset{k}{\text{min}\;}\left|q_{j}-{\hat{q}}_{k}\right|,\underset{k}{\text{max}}\underset{j}{\text{min}}\left|q_{j}-{\hat{q}}_{k}\right|\right\},$$

where$n_{s}$is the length of the largest segment,${\hat{q}}_{k}$are the estimated change points, and$q_{j}$are the true change points. The objective is to find a model that minimizes the scaled Hausdorff distance.

For each simulation, we generated 100 iterations. For FabiSearch: we used the R package NMF (Gaujoux & Seoighe, 2010) to implement NMF, the generalized Kullback–Leibler Divergence (1) for the loss measure, a minimum distance between change points ofδ=35, the number of runs$n_{\text{run}}\text{​}=50$, the number of permutations$n_{reps}\text{​}=100,$and a significance level ofα=0.01for the inference step inSection 2.3.3. For the simulations, we assume the number of clusters (or latent factors) is unknown, hence, we did not pre-specify rank and thus let FaBiSearch find the optimal value for the rank,r.

We compare our FaBiSearch method with the Network Change Point Detection (NCPD) method (Cribben & Yu, 2017) as it is the only competing high-dimensional method that has software available (which is not the case for the majority of the methods mentioned in the Introduction). NCPD finds change points in the network (or community) structure of multivariate high-dimensional time series data. Unlike FaBiSearch, NCPD uses binary segmentation and applies spectral clustering to find the communities based on the computed correlation matrix. The difference between the communities before and after a candidate change point is calculated using the principal angle, and a stationary bootstrap is used for inference. In the simulations, for NCPD, we used a pre-specified number of clusters,K, of one greater than the actual ($K_{0}+1$) as suggested in the paper, 1000 stationary bootstrap replications, a minimum distance between change points ofδ=50, and a significance level ofα=0.05. These are the default settings of the method, and note that the two methods use differentδ= 35 andδ= 50. We also compare with a more recent method fromBaek et al. (2023)which is PCA based and uses binary segmentation, which we refer to as PCAbinary for short. We use the detectR package (https://cran.r-project.org/package=detectR) to implement this method. PCA binary was designed to allow for temporal dependencies in the data. However, we found that PCA binary had an inferior performance when the data were not pre-whitened. Hence, we show the whitened results with superior performance for fairness.

#### 2.5.2 Simulated data

We now describe the simulated data. We chose simulations to emulate qualities of fMRI time series data. While the data are simulated from various models, we display the structure between the time series (or nodes) using graphs (or networks, which we use interchangeably) inFigure 4. A graph consists of a set of nodesNand corresponding edgesEthat connect pairs of nodes. Here, each node represents a time series, or ROI, and edges encode dependencies. In the fMRI setting, a missing edge indicates a lack of functional connectivity between corresponding regions. InFigure 4, the time series are represented by nodes and the colors of the nodes indicate cluster (or community) membership.

**Fig. 4.**
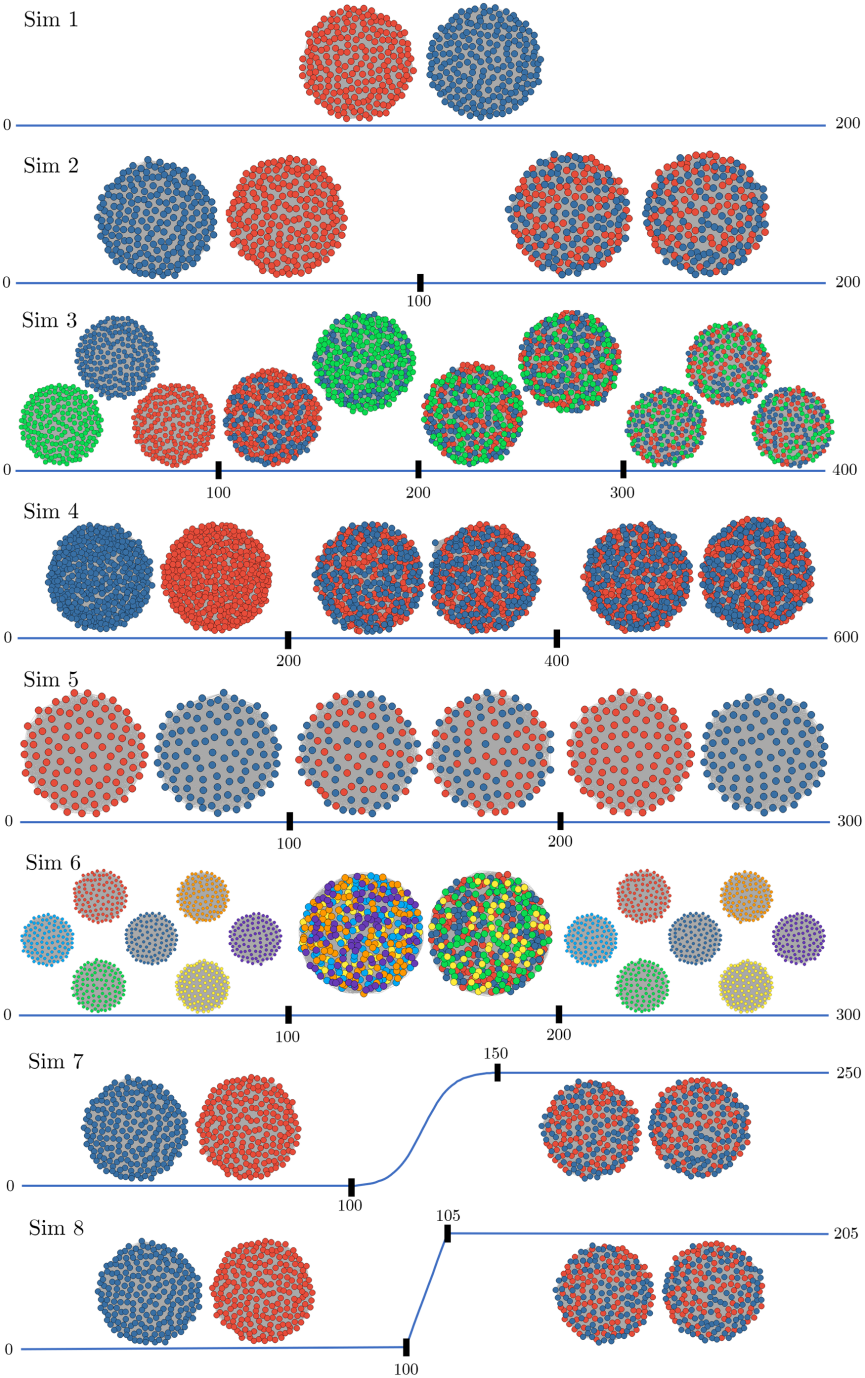
The true network structure of the simulations. The time series are represented by nodes. The colors of nodes indicate how the cluster membership evolves throughout the time series. Solid black vertical lines on the time axes indicate the location of the true change points. For Simulations 7 and 8, the slowly transitioning simulations, the shape of the change segments (100:150 and 100:105, respectively) denotes the weighting (sigmoidal and linear, respectively) of the clustering structure before and after the segment. Note that Simulations 9 and 10 use the same cluster structure as Simulation 2.

We progressively increased the difficulty and complexity of the simulations in order to characterize the power of FaBiSearch. All simulations are multivariate high-dimensional time series with a defined clustering (or community) structure, where$K_{0}$is the true number of clusters/communities.

Each simulation has stationary segments in which the data are generated from the multivariate Gaussian distributionN(0,Σ), where

$$\Sigma_{ij}=\{\begin{array}{cc} 0.75, & \text{if}\;i\neq j\;\text{and}\;i,j\;\text{are in the same cluster};\\ 1, & \text{if}\;i=j;\\ 0.20, & \text{otherwise}. \end{array}$$

**Simulation 1:**There are no change points.T=200,p=400time series, and the number of clusters is$K_{0}=2$. This simulation is similar to a steady-state fMRI time series, where the network structure does not change over time.

**Simulation 2:**We consider one change point.T=200,p=400, and the number of clusters is$K_{0}\text{​}=2$. The change point occurs at$q^{*}\text{​}=100$. Data before the change point are generated from the multivariate Gaussian distribution, where data after the change point have the node labels randomly reshuffled.

**Simulation 3:**We consider three change points.T=400,p=600time series. The change points occur at$q^{*}\text{​}=\left\{100,200,300\right\}$. In the first time segment, the true number of clusters$K_{0}\text{​}=3$, one of which is equally merged into the other two clusters at the first change point. Vertex labels are randomly shuffled at the second change point, while keeping$K_{0}\text{​}=2$. The true number of clusters$K_{0}$returns to 3, by moving one-third of each cluster into a new, third cluster.

**Simulation 4:**We consider two change points.T=600,p=800time series. The change points occur at$q^{*}\text{​}=\left\{200,400\right\}$. The number of clusters is$K_{0}\text{​}=2$throughout. At both change points, half of the vertices in each cluster are chosen at random and moved to the other cluster.

**Simulation 5:**We consider two change points.T=300,p=200time series. The change points occur at$q^{*}\text{​}=\left\{100,200\right\}$. The number of clusters is$K_{0}\text{​}=2$throughout. In the second time segment, the vertex labels are randomly shuffled from the first time segment. In the third time segment, the vertex labels are the same as the first time segment. In this simulation, we are mimicking the ABA structure, where the subject alternates between two states.

**Simulation 6:**We consider two change points.T=300,p=200time series. The change points occur at$q^{*}\text{​}=\left\{100,200\right\}$. In the first and third time segments,$K_{0}\text{​}=7$and vertex labels are the same. In the second time segment, the vertex labels from clusters 1 to 3 and 5 to 7 in the first time segment are grouped into two larger clusters. Half of vertex labels from cluster 4 in the first time segment are put into each of these two larger clusters. In this simulation, we are mimicking the ABA structure, where the subject alternates between two states. The number of clusters match the real fMRI data considered inSection 3.3.

**Simulation 7:**We consider one change point.T=200,p=400time series. For the first stationary segment, (1:100), data are generated from the multivariate Gaussian distributionN(0,Σ), whereas in the second stationary segment (150: 250) vertices labels are randomly reshuffled. The change segment occurs at$q^{*}\text{​}=100\;:150$, which is a weighted combination of stationary segments 1 and 2. Weighting is defined by$w_{c}=\frac{1}{1+e^{-0.2*\left(t-25\right)}}$, wheretis the number of time points after100.$K_{0}\text{​}=2$. In this simulation, we are mimicking a very slow transition between states.

**Simulation 8:**We consider one change point.T=200,p=400time series.$K_{0}\text{​}=2$. For the first stationary segment, (1 :100), data are generated from the multivariate Gaussian distributionN(0,Σ), whereas in the second stationary segment (105 :205) vertices labels are randomly reshuffled. The change segment occurs at$q^{*}\text{​}=100\;:105$, which is a weighted combination of stationary segments 1 and 2. Weighting is defined by$w_{c}=\frac{t}{10}$, wheretis the number of time points after100. In this simulation, we are mimicking a transition between states.

**Simulation 9:**We consider one change point.T=200,p=400, and the number of clusters is$K_{0}\text{​}=2$. The change point occurs at a random time point betweent=50and150for each iteration of the simulation. Data before the change point are generated from the multivariate Gaussian distribution, where data after the change point have the node labels randomly reshuffled.

**Simulation 10:**We consider one change point.T=200,p=400, and the number of clusters is$K_{0}\text{​}=2$. The change point occurs at$q^{*}\text{​}=100$. Data before the change point are generated from the multivariate Gaussian distribution, where data after the change point have the node labels randomly reshuffled. We add autocorrelation to these data using an AR(1) process,$X_{t}=\phi_{1}X_{t-1}+{\#}_{t}$, with$\phi_{1}\text{​}=0.7$.

Simulations 2, 3, and 4 were taken directly fromCribben and Yu (2017), for comparison purposes.

#### 2.5.3 fMRI study setup

We also applied FaBiSearch to two fMRI data sets: a resting-state fMRI data set and a task-based fMRI data set. NMF, by definition, requires the input matrix to be non-negative, however, fMRI data have no such restriction. To circumvent this issue, we shift the data to make it positive by adding the same positive value to all entries of the input matrix. This ensures that the input matrixXcontains only positive values while also preserving individual variability and the covariance between ROIs. We used the same inputs for FaBiSearch for analyzing the fMRI data set as in the simulation study (beginning ofSection 2.5.1).

#### 2.5.4 Resting-state fMRI data

This data set includes 25 participants (mean age of 29.44±8.64 years, 10 males and 15 females) scanned at New York University over 3 visits (http://www.nitrc.org/projects/nyu_trt). For each visit, participants were asked to relax, remain still, and keep their eyes open. A Siemens Allegra 3.0-Tesla scanner was used to obtain the resting-state scans for each participant, however, we considered only the second and third visits because they were less than an hour apart. Each visit consisted of 197 contiguous EPI functional volume scans with time repetition (TR) of 2000 ms, time echo (TE) of 25 ms, flip angle (FA) of 90°, 39 number of slices, matrix of64×64, field of view (FOV) of 192 mm, and voxel size of3×3×3mm^3^. Software packages AFNI (http://afni.nimh.nih.gov/afni) and FSL (http://www.fmrib.ox.ac.uk) were used for preprocessing. Motion was corrected using FSL’s mcflirt (rigid body transform, cost function normalized correlation, and reference volume the middle volume). Normalization into the Montreal Neurological Institute (MNI) space was performed using FSL’s flirt (affine transform, cost function, mutual information). Probabilistic segmentation was conducted to determine white matter and cerebrospinal fluid (CSF) probabilistic maps and was obtained using FSL’s fast with a threshold of 0.99. Nuisance signals (the six motion parameters, white matter signals, CSF signals, and global signals) were removed using AFNI’s 3dDetrend. Volumes were spatially smoothed using a Gaussian kernel and FWHM of 6 mm with FSL’s fslmaths. We used the work ofGordon et al. (2016)to determine the ROI atlas. The cortical surface is parcellated into 333 areas of homogeneous connectivity patterns, and the time course for each is determined by averaging the voxels within each region for each subject. Regional time courses were then detrended and standardized to unit variance. Lastly, a fourth-order Butterworth filter with a 0.01–0.10 Hertz pass band was applied. The final multivariate time series for the resting-state data contained 2 visits, each withT=197time points andp=333ROIs, for each of the 25 subjects (more details are available inAppendix A).

#### 2.5.5 Task-based fMRI data

This data set includes 8 participants (ages 18–40 years) scanned at the Scientific Imaging and Brain Imaging Center at Carnegie Mellon University (Wehbe et al., 2014;http://www.cs.cmu.edu/~fmri/plosone/). Subjects in the study were asked to read Chapter 9 of*Harry Potter and the Sorcerer’s Stone*(Rowling, 2012). All subjects had previously read the book or seen the movie. The words of the story were presented in rapid succession, where each word was presented one by one at the center of the screen for 0.5 s in black font on a gray background. A Siemens Verio 3.0T scanner was used to acquire the scans, utilizing a T2* sensitive echo planar imaging pulse sequence with repetition time (TR) of 2 s, time echo (TE) of 29 ms, flip angle (FA) of 79°, 36 number of slices, and3×3×3mm^3^voxels. Data were preprocessed as described inWehbe et al. (2014);Xiong and Cribben (2022). For each subject, functional data underwent realignment, slice timing correction, and co-registration with the subject’s anatomical scan, which was segmented into gray and white matter and cerebrospinal fluid. The subject’s scans were then normalized to the MNI space and smoothed with a 6× 6× 6 mm Gaussian kernel smoother. Data were then detrended by running a high-pass filter with a cutoff frequency of 0.005 Hz after being masked by the segmented anatomical mask. Finally, the Gordon brain atlas (Gordon et al., 2016) was again used to extract ROIs. The final time series for the task-based data contained 4 runs (324, 337, 264, and 365 time points) of 333 ROIs for each subject (more details are available inAppendix A).

## 3 Results

### 3.1 Simulation results

In this section, we present the simulation and fMRI results. In both simulations and fMRI data, we assumed for FabiSearch that the number of clusters (or latent factors) is unknown and has to be estimated in each case, while for NCPD we assumed a pre-specified number of clusters,K, of one greater than the actual ($K_{0}+1$), hence optimizing more in favor of NCPD. We evaluate the change point results by calculating the true and false positives within two margins (±10and±1time points). An FP rate of 1 indicates that 1 FP change point is detected in all the 100 simulations, on average. Hence, an FP rate greater than 1 is possible as more than 1 FP change point can be detected across the entire time course.

**Appendix Fig. A1.**
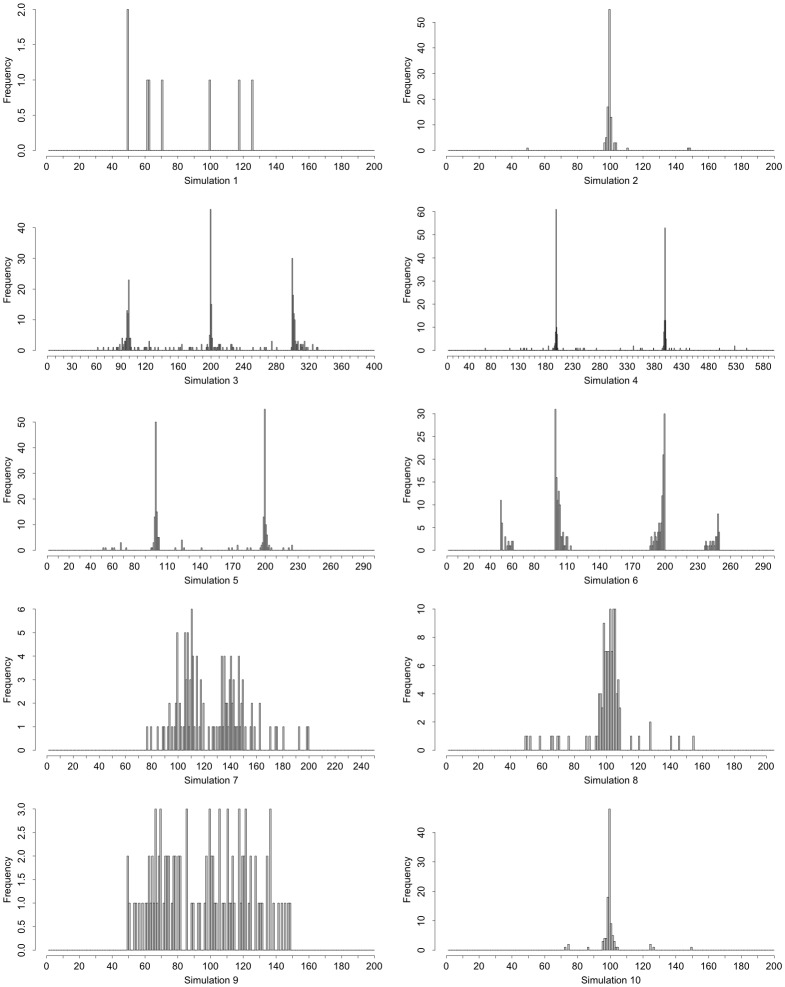
The histograms of the detected change points from FaBiSearch over 100 iterations for each simulation.

Overall, across the simulations, the FaBiSearch method outperforms the competitor NCPD (Cribben & Yu, 2017) with the results displayed inFigure 5(and inAppendix Table A4in theAppendix A). More specifically, in Simulation 1, where there are no change points, FaBiSearch obtains a small false positive (FP) rate of 0.08, compared with NCPD’s 0.89. In Simulation 2, the two methods perform similarly on detecting one change point. FaBiSearch obtains a true positive (TP) change point rate of 0.99 across the 100 iterations while NCPD achieves a TP rate of 1, and the FP rates are the same. However, once the number of time series and the complexity increase, FaBiSearch establishes a clear advantage over NCPD. For example, in Simulation 3, FaBiSearch finds 54%more true change points than NCPD while having a reduction of 34%in the number of FPs compared with NCPD. The improvement of FaBiSearch over NCPD increases further in Simulation 4, where FaBiSearch finds 1.7 times more TP change points while reducing the number of FPs to 9%of those detected by NCPD. In Simulation 5, we tested the two methods on an “ABA” type structure in which the first and last clustering structures are identical. This makes the change points less discernible and thus change point detection more difficult, especially for binary segmentation methods (given the similarity between any two partitions). FaBiSearch, however, performs well by detecting 93.5%of the true change points, outperforming NCPD again while detecting only a fraction of the false positives. Simulation 6 has a similar structure albeit with 7 clusters instead of 2 in the first and final stationary segments. Here, FaBiSearch outperforms NCPD again on all accounts; NCPD was not able to find any change points. FaBiSearch finds 95%of the true change points while obtaining an FP frequency of just 0.69. In Simulation 7, where there is one change segment from 100 to 150 (the network is slowly transitioning between two states), FaBiSearch and NCPD perform similarly in TP rates, obtaining frequencies of 1. FaBiSearch, however, obtains a smaller FP frequency of 0.13 compared to NCPD’s 0.79. A similar outcome is seen in Simulation 8, where the change segment is linearly weighted across five time points (the network is slowly transitioning between two states). Here, FaBiSearch outperforms NCPD with a TP rate of 0.93 compared with 0.16, and the FP rate favors FaBiSearch (0.17) over NCPD (0.69). Overall, FaBiSearch finds the true change points more frequently, while drastically reducing the number of FPs compared to NCPD. Finally, it is important to point out that the dimensionality of the problem is large, hence making the detection difficult as indicated by the performance of NCPD. Across all simulations except Simulations 1 and 7, we find that the Hausdorff distance for FaBiSearch is lower than for NCPD, (seeAppendix Table A4in theAppendix A), indicating that the change points detected by FaBiSearch are closer to the true change points, hence, FaBiSearch outperforms NCPD on another metric. Finally, it appears that Fabisearch does not often find change points at the edges of the time series, unlike NCPD (Appendix Fig. A1inAppendix Ashows histograms of the detected change points for FaBiSearch in the simulations).

**Fig. 5.**
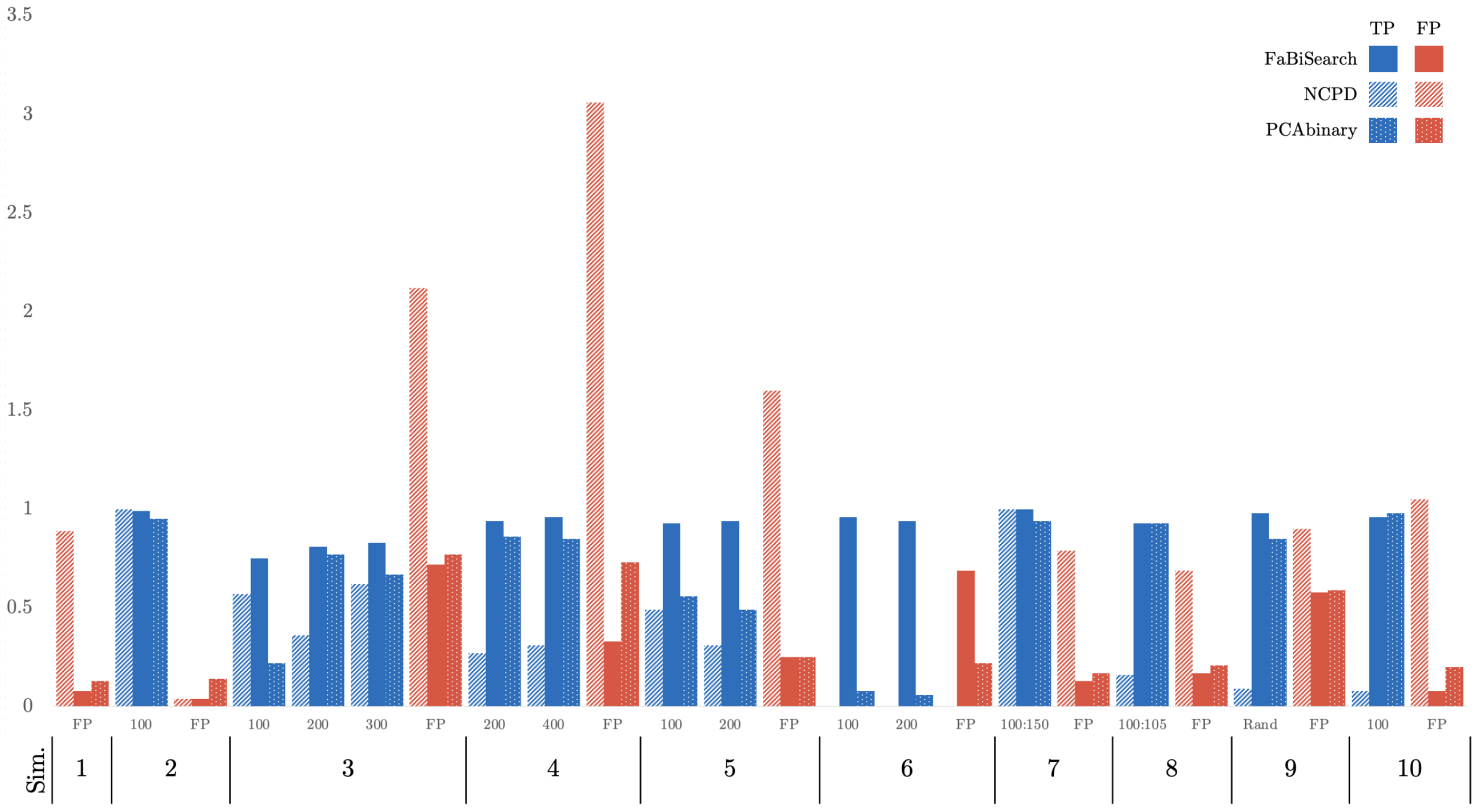
A plot of the true positive (blue bars) and false positive (red bars) rates for NCPD (striped fill) and FaBiSearch (solid fill) across all the simulations using the±10window. Thex-axis represents the simulation number and they-axis represents the TP (blue bars) and FP (red bars) rates. The tables for both the±10and the±1windows can be found in theAppendix A.

In comparison to another state-of-the-art method, FaBiSearch also generally outperforms PCAbinary across all simulations. For PCAbinary, we use the default settings for change point detection using the asymptotic threshold method. For the no change point, one change point and multiple change points settings (Simulations 1–6), FaBiSearch dominates PCAbinary, across all accuracy metrics, TP, FP and Hausdorff distance. This is especially evident in the multiple change point settings. In the smoothly transitioning settings (Simulations 7 and 8), the performance of FaBiSearch and PCAbinary is similar. In Simulation 7, both methods had a TP1 of 0.94, but FaBiSearch had a slightly higher FP1 of 0.24 to PCAbinary’s 0.21; however, FaBiSearch achieved a perfect TP10 of 1.0 versus 0.94 for PCAbinary. In Simulation 9 with randomly placed change points, FaBiSearch outperformed PCAbinary with a TP1 of 0.65 versus 0.56, and a lower Hausdorff distance (0.3523 vs. 0.3942). Finally, in Simulation 10 with$q^{*}\text{​}=100$, PCAbinary slightly outperformed FaBiSearch in TP1 (0.80 vs. 0.75) and had a lower Hausdorff distance (0.015 vs. 0.0244), though FaBiSearch had a lower FP1 (0.29 vs. 0.38). Overall, these results suggest that FaBiSearch generally provides higher true positive rates and lower false positive rates across simulations, indicating more accurate change point detection than PCAbinary.

We also assessed the efficacy of NMF in estimating networks between each pair of change points (or stationary block) in the simulations. To this end, for each iteration of each simulation, we first detected the change points and hence each stationary block using FaBiSearch. Then for each stationary block, we estimated the network using our method described inSection 2.4where we specified the true number of clusters,$K_{0}$, as the cutoff point for the resulting tree. Since these adjacency matrices are symmetric, we quantified the difference between the true adjacency matrix and the NMF calculated adjacency matrix by calculating the percent overlap of the off-diagonal elements. The results are shown inAppendix Table A1(inAppendix A). The method performs well and can recover the true network structure between change points across all simulations.

### 3.2 Resting-state fMRI results

**Fig. 6.**
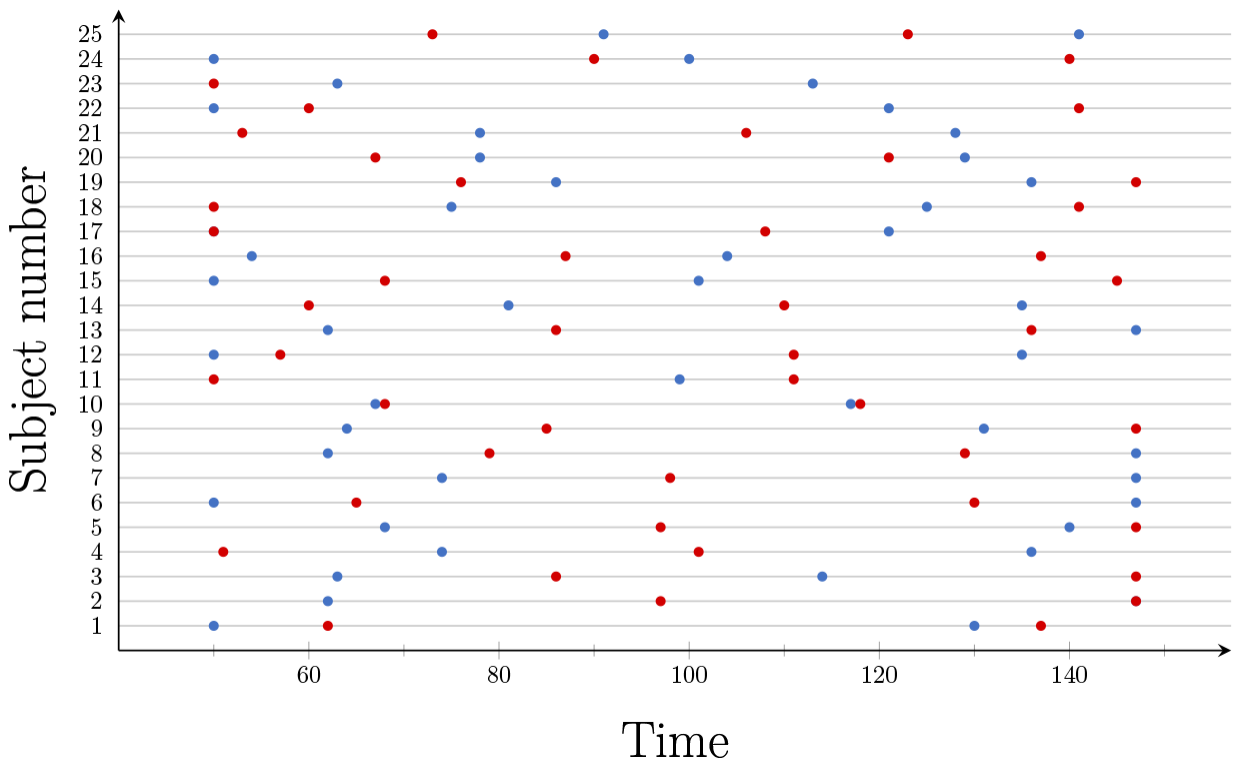
The detected change points for each subject in the resting-state fMRI data set. Blue and red dots denote change points for the second and third scans, respectively.

The objective of applying FaBiSearch to these data is to study the test–retest reliability and behavior of dynamic FC, hence we only consider the second and third scans, which were obtained less than an hour apart (Section 2.5.4). We did not consider the first scan as it was taken 5–11 months before the second resting-state scan.Figure 6shows the detected change points for the resting-state fMRI data set using FaBiSearch. FaBiSearch has been applied to each subject and scan combination separately. Blue and red dots denote the change points from the second and third scans, respectively. Although we are not certain of the number of change points for a resting-state fMRI experiment, as the subject is in an unconstrained state, we expect subjects to drift between different states (or functional modes), which is consistent with previous work (Anastasiou et al., 2022;Cribben & Yu, 2017;Delamillieure et al., 2010;Doucet et al., 2012). We found that each subject has a unique set of change points. Comparing the results across scanning sessions and between subjects, we see variability in the location and number of detected change points.Xu et al. (2021)analyzed these data to study the test–retest reliability of static FC, however, here we are considering the test–retest reliability of dynamic FC. Some subjects (e.g., subject 14) have quite different change points across the scanning sessions, while others (e.g., subjects 1, 10, 19, 20, 21, 22) have change points that are consistent across the two scans.

**Fig. 7.**
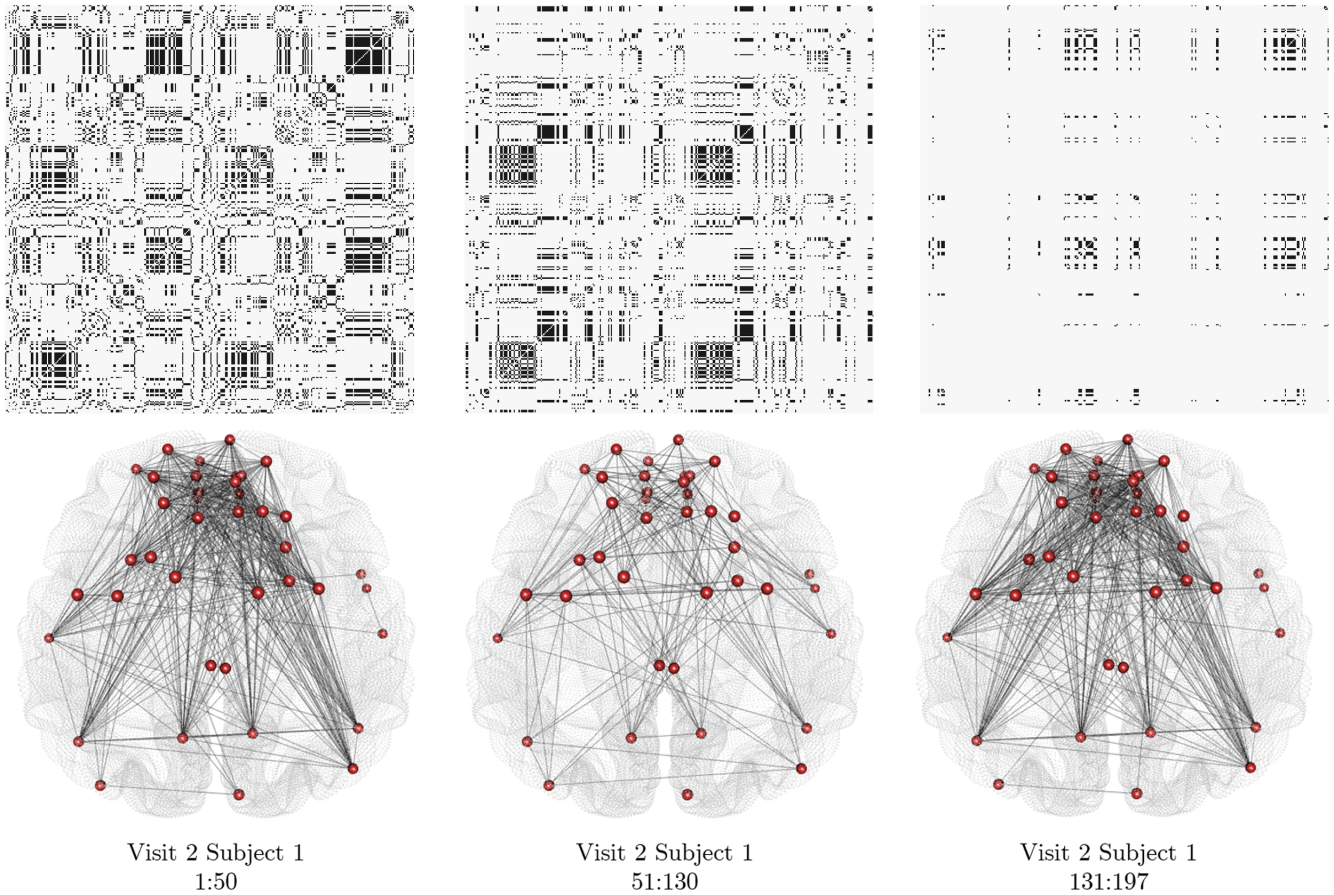
The stationary networks between each pair of change points for subject 1’s second resting-state fMRI data. The top panel shows the adjacency matrices, where the x and y axes correspond to the 333 ROIs from the Gordon atlas, whereas the bottom panel networks are for only the “Default” community.

To visualize and interpret the network structure between each pair of change points detected by FaBiSearch, we estimate the networks using the NMF cluster-based method described inSection 2.4. For this procedure, we fixed the number of runs ($n_{\text{runs}}=100$) to calculate the consensus matrix. Previous work (Allen et al., 2014) identified seven unique resting-state networks, hence we used this prespecified cluster size.Figure 7depicts the stationary networks between each pair of change points for subject 1’s second fMRI resting-state scan. The networks allow us to compare the intrasubject community structure. There is a clear time-varying relationship between the ROIs within this network. In fact, there are substantial changes in the clustering structure across the segments, with the first network in the top panel having a strong block diagonal structure and this progressively diminishes over the subsequent segments. Additionally, the density of the overall networks diminishes over time. In comparison, however, in the bottom panels where only edges in the “Default” community are plotted, the density appears to be high, then decreases, and then returns to high again. The first and third networks in the bottom panel appear quite similarly connected (similar to an ABA type structure, see the simulations inSection 2.5.1), with extremely dense and abundant connections between most of the nodes, and very few connections to the two nodes near the periphery of the rear and the three nodes on the right periphery. This is in contrast to the second network in the bottom panel, where the peripheral nodes, especially the three on the right are more interconnected with the rest of the nodes. These results provide evidence that the subject’s FC, and, therefore, mental state, was evolving during rest. Furthermore, it also suggests that the overall networks and individual communities evolve in an independent manner.

InFigure 8, we compare the stationary networks in the “Default” community across both subjects and scans. There is a clear presence of a common stable network (or subnetwork) or motifs. This suggests that there may exist certain motifs or sub-networks in time-varying FC that remain stable across subjects. Since these motifs appear across different stationary segments, it suggests that the resting-state networks do not evolve in an identical manner across subjects, as during a resting-state experiment, the subjects are unconstrained.

**Fig. 8.**
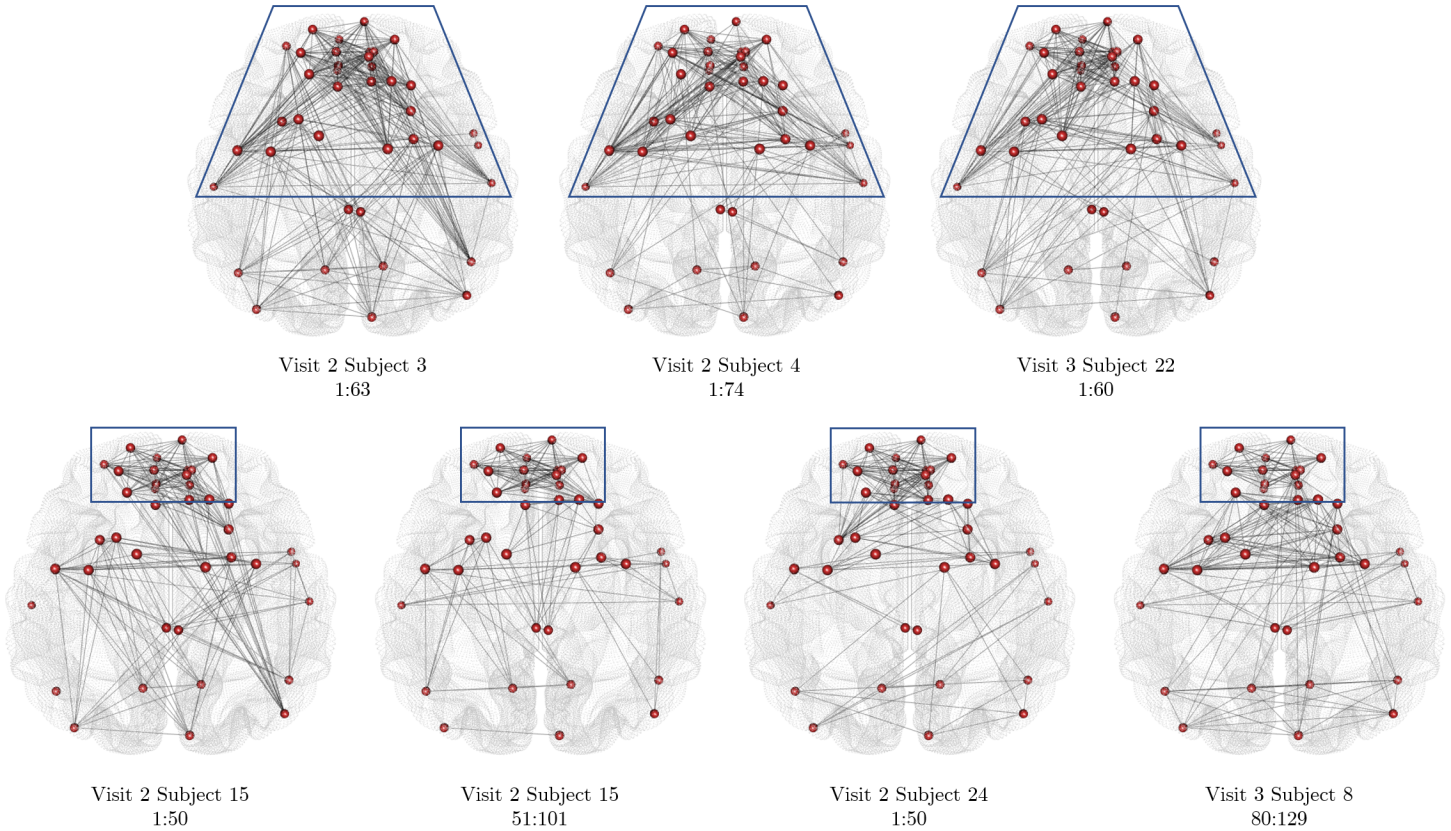
The estimated networks for different stationary segments across subjects and scans for the resting-state fMRI data. Similar network motifs are outlined in blue across both top and bottom panels.

### 3.3 Task-based fMRI results

**Fig. 9.**
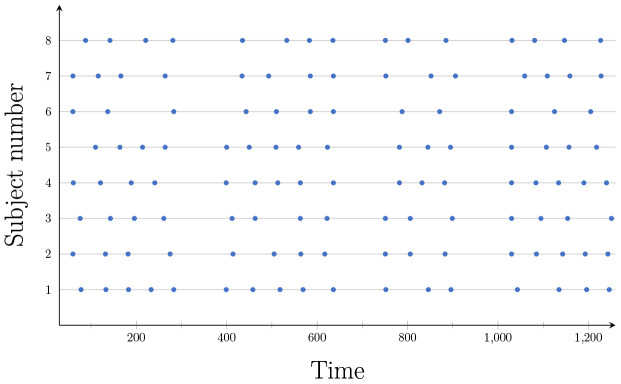
The detected change points for each subject in the task-based fMRI data set. Blue dots denote change points, and runs 1–4 are concatenated for each subject.

Figure 9shows the detected change points for the task-based fMRI data set (Section 2.5.5) using FaBiSearch. The results are concatenated across runs for each subject, which explains the presence of gaps, where no change points were identified. Overall, many change points were detected. In addition, there is some variability in the location and number of detected change points across subjects. Intuitively, as we considered a large number of ROIs (p=333) and as reading is a complex and involved task, we expect FC to be constantly evolving and different for each subject based on unique factors such as age, interpretation of the story, and familiarity with the text. There are some change points, however, which are consistent across subjects, especially at critical moments and events in the storyline.

**Fig. 10.**
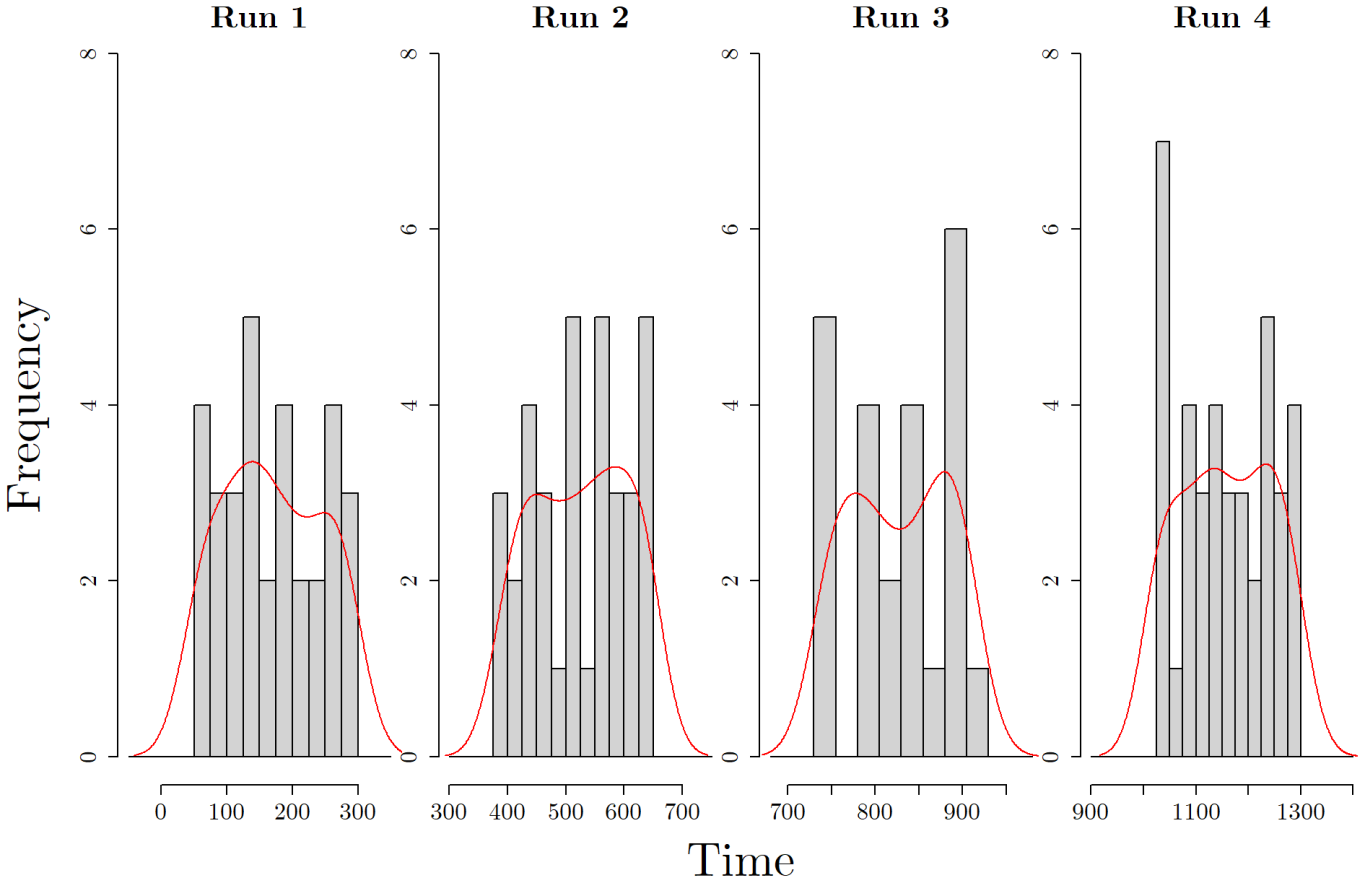
The histograms of the detected change points in the task-based fMRI study across all subjects (gray bars), overlaid with density estimates using a Gaussian kernel (red lines).

Figure 10shows the histograms of the change points concatenated across subjects. Given there are only eight subjects in this study, the histograms are very sensitive to bin size, hence we have overlaid density estimates for each of the four runs. In particular, we seek to find density estimates which show concentrated density and thus indicate a general pattern across subjects. We see that some of the smoothed peaks coincide with major events in the story. In the first run, the smoothed plot shows a peak at time pointt=139, which coincides with Draco taking Neville’s remembrall, and precedes the first flying lesson for Harry and his classmates. The second run coincides with the following story line: after the class is left unattended by the teacher, Madam Hooch, Harry chases classmate Draco Malfoy on a broom, having never flown before. Another teacher, Professor McGonagall, spots this mischief and, instead of punishing Harry, offers him a spot as a Seeker on the Gryffindor Quidditch team. The density peak for this run is at approximately time pointt=585, and coincides with this offer, marking the beginning of Harry’s Quidditch playing career, which is an important recurring narrative in the book series. In the third run, Harry has accepted a wizard’s duel that night with arch-rival Malfoy and is on his way with Ron to meet Malfoy at the trophy room. The change points detected peaks at two time points, where the first att=778marks a time point just after Hermoine warns Harry and Ron not to go through with the wizard’s duel. The second peak, at time pointt=879,occurs after Hermoine surprises Harry and Ron by catching them, and scolding them for trying to break school rules. In the fourth run, the density peaks noticeably at time pointt=1233. In this part of the story, the three of Harry, Ron, and Hermoine are almost caught by Argus Filch, the Hogwart’s caretaker, and his cat, Mrs. Norris. As the children run, they get lost and end up in a forbidden area on the third floor. The peak seen att=1233coincides with the children running after meeting a scary and large three-headed dog.

**Fig. 11.**
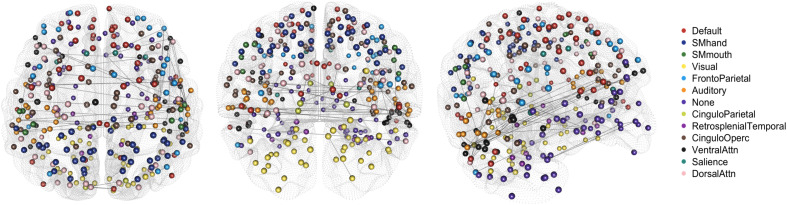
The aggregated network composed by combining all the stationary networks of all subjects. An edge is included in the network if it is estimated>17times (99.9th percentile), based on the sum of edges in adjacency matrices across the stationary networks of all subjects. The aggregate network is shown at three different angles, from the superior in the transverse plane, rostral in the coronal plane, and between the coronal and saggitial planes, respectively.

We also examine the stationary networks between change points. Given we have no*a priori*knowledge of the number of clusters for this data set, we use the cutoff-based method to estimate these networks (Section 2.4). We choose a cutoff value which equates to approximately 100 edges. This provides a high level of sparsity in the networks to help interpretability.Figure 11shows the aggregate network across subjects. This network is created by first running FaBiSearch on all subjects separately, estimating stationary networks between each pair of change points from each subject, and then combining all these networks into one network. This process involves aggregating information across 161 stationary networks. An edge is present in the aggregated network if it occurs with a frequency greater than 17 (99.9th percentile). In the aggregated network, many edges interconnect the left and right hemispheres of the brain. Additionally, there is some evidence of lateralization of connectivity in the frontal section of the right hemisphere. Furthermore, the nodes which have a high degree are concentrated in the right hemisphere. Nodes with degree>5,>10, and>20are in the right hemisphere51.0%,54.5%, and58.5%of the time, respectively; nodes with more “hub”-like behavior are more likely to appear in the right hemisphere. While reading and language comprehension are distributed processes which involve many regions of the brain (Price, 2012), it has been suggested that the right hemisphere is associated with integrating and combining information into higher level processes such as perception and understanding (Mashal et al., 2005;St George et al., 1999) with the more basic processes such as word recognition, syntax, and semantics typically associated with the left hemisphere (Binder et al., 2003;Friederici, 2002).

The five highest degree nodes (in decreasing order, 232, 225, 68, 239, and 226) in the aggregated network (Fig. 11) belong to the ventral attention, default, and auditory communities from the Gordon atlas, which play an important role in executing temporally stable operations in the otherwise dynamic reading process. For example,Seghier and Price (2012)suggested that the default mode community has various roles during semantic processing, whileCorbetta and Shulman (2002)suggest that components of the ventral attention community are important for “orienting” attention, especially when presented with unexpected novel or stimuli. The latter suggests that the ventral attention community could be activated by mediating the changes in DFC network structure which are a function of the underlying changes in the story/narrative. We also see that some nodes are consistently interconnected with a high degree in the auditory community (p=24). This supports the work ofYao et al. (2011)who discovered that the auditory cortex is strongly activated during silent reading, indicative of auditory imagery—or having an “inner voice” while reading. Moreover, the aforementioned high-degree nodes are located specifically in Brodmann area 22, which contains components of Wernicke’s area (Binder, 2015) which has long been associated with language comprehension. Thus, it is understandable, from a network perspective, that the nodes related to this area are of high degree and have strong connections to other brain regions during reading. Although reading is a complex task, and people’s individual experiences with and understanding of the text play a role in their FC, commonalities do exist. The collection of estimated networks suggests reading is a highly dynamic and integrated process, where more lower level FC features remain stable but higher level processes and patterns are highly variable and different across subjects.

**Fig. 12.**
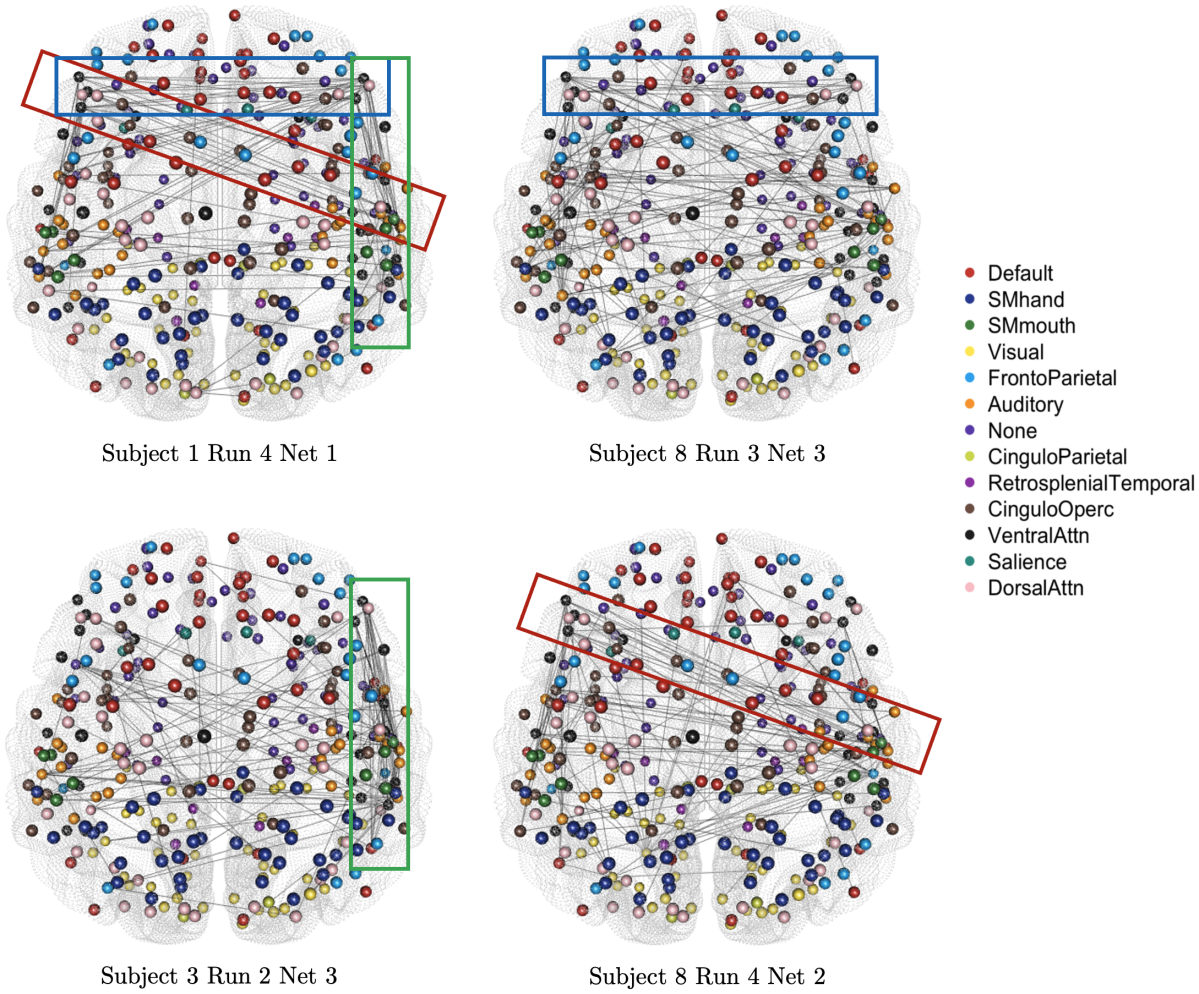
The estimated stationary networks comparing motifs for subject 1 run 4 network 1 (top left), subject 8 run 3 network 3 (top right), subject 3 run 2 network 3 (bottom left), and subject 8 run 4 network 2 (bottom right) for the task-based fMRI study.

We also explore the network characteristics across the stationary networks both within and between subjects.Figure 12shows similar network motifs in the first stationary network for subject 1 run 4, and the stationary networks for different subjects and stationary blocks. The first motif (outlined in blue) shows strong cross-midline connectivity between frontal brain nodes, with edges predominantly perpendicular to the midline. The next is somewhat of a pivot, outlined in red, where nodes in the front left area of the cortex are densely connected to nodes in the middle area on the right, close to the temporal region. The last motif, outlined in green, is localized to the right side of the brain, where edges are strongly interconnected all across the outer right side of the cortex. The individual networks are different across subjects, runs, and stationary segments, however, the presence of these motifs suggests similarities and stability for network characteristics and FC patterns.

**Fig. 13.**
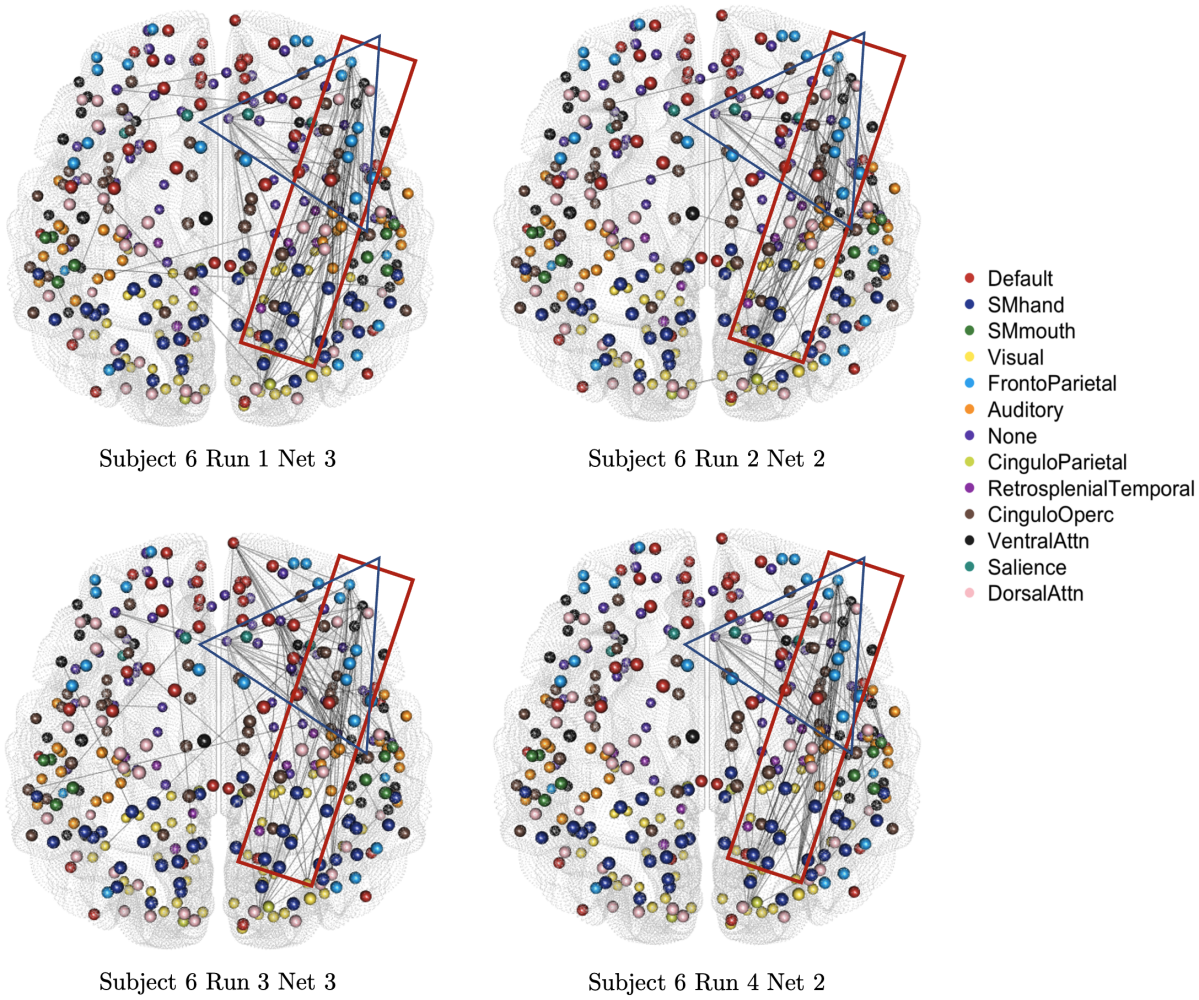
The estimated stationary networks for subject 6 comparing motifs across different runs and stationary networks for the task-based fMRI study.

Within subjects, there are also some similar patterns across stationary networks.Figure 13shows the similar network features for subject 6 across a variety of different runs and stationary segments. These motifs, or subnetworks, again indicate some stability in the temporal relationships between nodes. These individual networks are still unique, as expected, given in each of these segments the subject was reading a different part of the story, and, therefore, it is logical that how they mentally process the story differs.

**Fig. 14.**
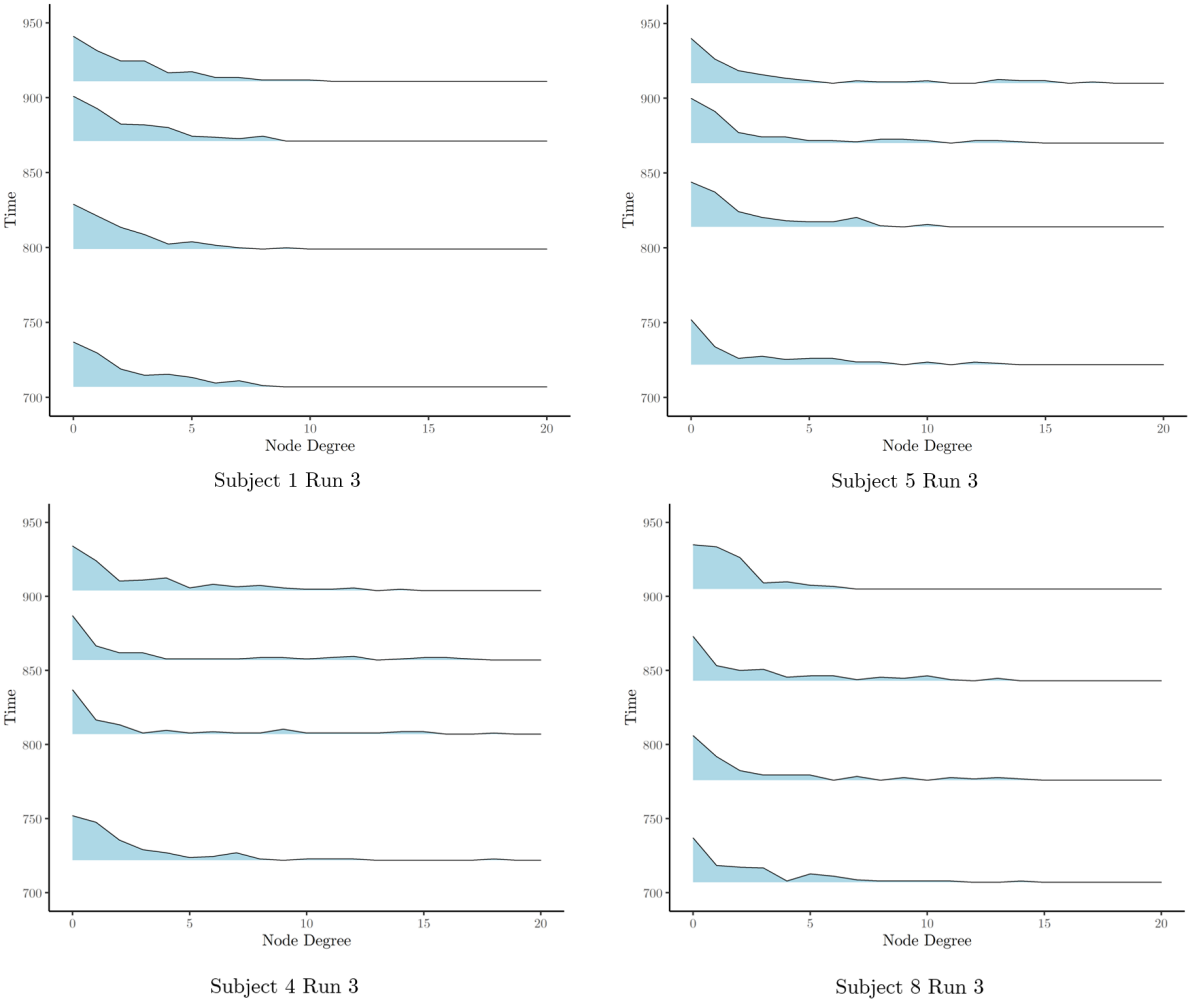
The degree distributions for the stationary networks in run 3 for subjects 1, 4, 5, and 8 of the task-based fMRI data set. Each degree distribution is centered between detected change points along the y-axis, and the heights are plotted according to a logarithmic scale to highlight the power law.

We next explore the degree distributions across subjects. In particular, we first run FaBiSearch on run 3 of the task-based fMRI data set for subjects 1, 4, 5, and 8. This run is the most dynamic in terms of story features and also has consistent change points across the chosen subjects. For each stationary network, we compute the degree distributions. InFigure 14, we plot the degree distribution, where each degree distribution is centered between detected change points along the y axis and the heights are plotted according to a logarithmic scale to highlight the power law. We find that all subjects’ individual networks obey the power law of degree distribution, wherein the majority of nodes have low degree and a minority have very high degree. These high-degree nodes are perceived as important “hub” nodes, which is an important and common feature observed in many real-world, including brain, networks. The distributions also seem to follow a similar temporal pattern. For example, for subjects 4 and 8, the distributions appear to move from heavy tailed to lighter tailed from the first stationary network to the next. Then, the second and third stationary networks have nodes with a degree>10, indicating greater “hub”-like behavior for these nodes. Lastly, the final degree distributions are more concentrated in the1−10degree range. Networks from other subjects and runs also obeyed the power law of degree distribution. However, subjects also vary in the temporal patterns in these distributions. For example, for subjects 1 and 5 (Fig. 14), the distributions appear to become more heavily tailed in the last two stationary segments, wherein these segments have node degrees comparatively more concentrated in the>10range. Furthermore, it is possible that the degree distributions and networks themselves may be related to the narrative of the story, or more broadly, the tasks that subjects are participating in as well as their individual interpretations and perspectives on the story.

## 4 Discussion

### 4.1 Extensions

Non-negative matrix factorization (NMF) is an integral component of FaBiSearch and the proposed graphical method. Our simulations and data analysis show that NMF is capable of creating a low rank approximation of the data and retain dominant network (or clustering) structures. For FaBiSearch, we concluded that the algorithms ofLee and Seung (2000)in combination with the generalized Kullback–Leibler divergence performed best for our simulations and fMRI data, however, we also considered other algorithms including the Alternating Least Square (ALS) approach, which minimizes an Euclidean-based objective function, that is regularized to favor sparse basis matrices or sparse coefficient matrices. Our choices provided a balance between specificity and sensitivity in all signals examined in our large-scale simulation study and others not included. Hence, all have been calibrated on higher dimensions. The practitioner has the option to use alternatives to these in our R package**fabisearch**(Ondrus & Cribben, 2021).

Furthermore, our proposed binary search method in FabiSearch is computationally faster than standard sequential searches, but is possibly limiting in that it is a purely greedy method. In particular, by iteratively halving the search space, binary search improves upon the sequential, exhaustive search of binary segmentation by reducing the worst-case search size fromO(T)toO(logT), whereTis the number of indices to be evaluated. More exploration into non-greedy methods might yield more favorable results and/or decrease computational load. We also intend to explore other change point segmentation methods such as isolate detect (Anastasiou et al., 2022). As an extension to monitoring change points in network objects, we intend to incorporate higher order structures, such as tensors, in the NMF procedure of FaBiSearch. Finally, we have shown that the residuals betweenXandW⋅Hin NMF contain important information about the clustering and dependence structure, however, it is likely there are alternative methods of extracting this information.

### 4.2 Computation

We compared FaBiSearch with another method, Network Change Point Detection (NCPD), and found that in the same simulations, FaBiSearch has a superior performance across all evaluation criteria. However, in comparison to NCPD, the computational complexity of FaBiSearch is greater. For example, in Simulation 1, using 48 core machines with 2 Intel Platinum 8260 Cascade Lake at 2.4 Ghz and 187 GB of memory, FaBiSearch took 25.04 min on average, while NCPD took 0.75 min on average across the 100 iterations. While the binary search algorithm in FabiSeach is computationally efficient, the method is hindered by the NMF algorithm and the permutation test. However, what is lost in computational speed is gained in accuracy.

To compare the binary search method in FaBiSearch with the binary segmentation method, we consider Simulation 5.Appendix Table A3(in theAppendix A) shows the results of the simulation and shows that the binary search in FaBiSearch is more accurate (higher TP rates, lower FP rate, and lower Hausdorff distance), as well as considerably more computationally efficient (lower compute time) than binary segmentation.

### 4.3 Limitations

We acknowledge that the sample sizes in both the task-based (8 participants) and resting-state (25 participants) fMRI studies are relatively small, which may impact the robustness of our findings. In general, for resting-state fMRI data, inter-subject variability remains a challenge, and in particular, for our task-based fMRI data set, detecting shared dynamic changes is constrained by the small cohort (Grady et al., 2021;Ren et al., 2017).

We also acknowledge that our fMRI results are sensitive to the specific pre-processing steps, as well as intra-scanner noise and variability. For each study, the same scanner was used, but, pre-processing and intra-scanner variability may contribute false positives in our analyses. With this in mind, future studies with larger sample sizes would help validate and extend the findings from these data sets.

Given our findings from the current study designs, we recommend that future studies explore alternative study designs, particularly for task-based fMRI, to further validate the methodology and strengthen the correspondence between detected change points and task events. Additionally, future research could leverage change point information for downstream applications, such as predicting participant cognitive states or identifying pathological patterns.

An important consideration in our method is the magnitude of the positive shift applied to ensure non-negativity. While this did not impact change point detection, we acknowledge the potential for distortions at extreme values. We, therefore, advise users to carefully assess preprocessing choices and consider a sensitivity analysis with varying shift values to evaluate potential effects.

## 5 Conclusion

In this paper, we characterize and implement a novel multiple change point detection method in the network structure between multivariate high-dimensional time series, called factorized binary search (FaBiSearch), and a method for estimating stationary network structures between detected change points. We assumed the number and location of the change points are unknown a priori. Our methods have several strengths and unique features. First, FaBiSearch scales well to multivariate high-dimensional time series data. This allows us to detect change points in high-dimensional cortical atlas parcellations such as that ofGordon et al. (2016)to characterize whole-brain dynamics. There exist very few other change point detection methods capable of handling these wide data sets and we show through simulations how FaBiSearch outperforms two of these methods.

Second, our graph estimation method finds only positive relationships between nodes. Unlike correlation measures, which can take any value in[−1,1], our new method is limited to[0,1]. This is because the consensus matrix, which is the basis for this new method, is constrained to[0,1]as it is the arithmetic average of connectivity matrices which are themselves limited to{0,1}. This makes the corresponding graph more intuitive and understandable, as anticorrelations in an FC context are difficult to interpret.

Lastly, non-negative matrix factorization (NMF) is an integral component of FaBiSearch and the graphical method. This is unique in two distinct ways. We are, to the best of our knowledge, the first to use NMF for change point detection, network estimation, and to apply it specifically to fMRI data. Second, we use NMF to discover dependence structure. While some methods use clustering and/or dimension reduction techniques, these are usually applied in conjunction covariance estimation (or correlation or precision matrices). FaBiSearch solely uses NMF as a method of finding a dependence structure among variables, with no intermediary method of finding dependence among variables.

We also applied FaBiSearch to a resting-state and a task-based fMRI data set. For the resting-state experiment, we analyzed the test–retest reliability of dynamic FC, while for the task-based experiment, we explored network dynamics during the reading of Chapter 9 in*Harry Potter and the Sorcerer’s Stone*. The large-scale characterizations of the FC structure have not been explored in these data sets before. In general, we detected many change points. This suggests that regardless of the fMRI study, the FC networks are constantly evolving. We further found common states both across and within subjects in both data sets. This is encouraging as it shows the stability of some features and networks across subjects in these studies. Naturally, FaBiSearch could also be used to determine novel biomarkers for neurological phenomena such as disease status or to find network structures corresponding to different thought processes or perceptions based on the subject-specific FC.

## Data Availability

The resting-state and task-based data sets were derived from the following public domainshttp://www.nitrc.org/projects/nyu_trtandhttp://www.cs.cmu.edu/~fmri/plosone/, respectively. The methodology is implemented in the R package fabisearch on CRAN (https://cran.r-project.org/package=fabisearch), as well as all experiments on GitHub (https://github.com/mondrus96/FaBiSearch_exp). For additional information regarding the software implementation as well as specific use cases, we refer readers toOndrus and Cribben (2024).

## Appendix A

### A.1. Simulation results

**Appendix Table A1.** For each simulation, a comparison of the true networks and the estimated networks using NMF between each pair of change points (stationary blocks).

Simulation	1	2	3	4	5	6	7	8
% Accuracy	100	100	99.16	99.69	99.68	96.1	98.84	99.95

The percentage was calculated as the proportion of overlap in the off-diagonal elements between the true adjacency matrix and the estimated NMF adjacency matrix.

### A.2. Experimental data dimensions

Appendix Table A2summarizes the dimensions of time series data for the resting-state and task-based fMRI experiments for each subjects’ visit.

**Appendix Table A2.** The number of time pointsTand number of variablespfor each subjects’ visit in the resting-state (RS) and task-based fMRI experiments.

Data set	T	p
RS Visit 2	197	333
RS Visit 3	197	333
TB Run 1	324	333
TB Run 2	337	333
TB Run 3	264	333
TB Run 4	365	333

**Appendix Table A3.** A comparison of binary segmentation and binary search used in FaBiSearch using simulated data from Simulation 5 albeit withp=20and completed over 10 iterations.

Search type	$t^{*}$	TP 1	FP 1	TP 10	FP 10	Compute mins.
segmentation	100	0.2	1.4	0.4	0.5	38.11
segmentation	200	0.2		**0.9**		
Binary	100	**0.6**	**0.9**	**1**	**0.2**	**3.93**
search	200	**0.5**		0.8		

Bolded values indicate the best performance for each metric across methods.

**Appendix Table A4.** The simulation results for FaBiSearch, NCPD, and PCAbinary.

		FaBiSearch						NCPD						PCAbinary					
	$q^{*}$	TP _1_	FP _1_	TP _10_	FP _10_	Haus.	$T_{\text{run}}$	TP _1_	FP _1_	TP _10_	FP _10_	Haus.	$T_{\text{run}}$	TP _1_	FP _1_	TP _10_	FP _10_	Haus.	$T_{\text{run}}$
1	NA	NA	**0.08**	NA	**0.08**	NA	16.41	NA	0.89	NA	0.89	NA	0.73	NA	0.13	NA	0.13	NA	**0.03**
2	100	0.85	0.18	0.99	**0.04**	0.0085	17.74	**0.88**	**0.16**	**1**	**0.04**	**0.0063**	0.39	0.71	0.38	0.95	0.14	0.0056	**0.04**
3	100	**0.39**	1.57	**0.75**	**0.72**	**0.1369**	84.93	0.23	2.98	0.57	2.12	0.3418	6.24	0.06	**1.47**	0.22	0.77	0.2678	**0.08**
	200	**0.66**		**0.81**				0.05		0.36				0.52		0.77			
	300	**0.49**		**0.83**				0.41		0.62				0.38		0.67			
4	200	**0.79**	**0.65**	**0.94**	**0.33**	**0.035**	268.97	0.27	4.27	0.58	3.6	0.2973	31.26	0.49	1.47	0.86	0.73	0.0952	**0.09**
	400	**0.79**		**0.96**				0.18		0.54				0.48		0.85			
5	100	**0.78**	**0.56**	**0.93**	**0.25**	**0.0468**	14.08	0.19	2.13	0.49	1.6	0.405	1.19	0.27	0.73	0.56	**0.25**	0.0834	**0.06**
	200	**0.78**		**0.94**				0.08		0.31				0.3		0.49			
6	100	**0.47**	1.61	**0.96**	0.69	**0.0425**	103.77	0	**0**	0	**0**	NA	0.57	0.02	0.34	0.08	0.22	0.486	**0.06**
	200	**0.51**		**0.94**				0		0				0		0.06			
7	100:150	**0.94**	0.24	**1**	**0.13**	0.1677	36.02	**1**	0.81	1	0.79	0.1068	1.33	**0.94**	**0.17**	0.94	0.17	**0.1257**	**0.04**
8	100:105	0.67	0.43	**0.93**	**0.17**	0.0615	23.49	0.05	0.8	0.16	0.69	0.339	0.71	**0.87**	**0.27**	**0.93**	0.21	**0.0448**	**0.04**
9	NA	**0.65**	**0.82**	**0.98**	**0.58**	0.3523	35.78	0.01	0.99	0.09	0.9	**0.225**	0.05	0.56	0.83	0.85	0.59	0.3942	**0.03**
10	100	0.75	**0.29**	0.96	**0.08**	0.0244	31.64	0.01	1.12	0.08	1.05	0.4358	0.16	**0.80**	0.38	**0.98**	0.20	**0.015**	**0.04**

Sim., mean rank,$q^{\text{*}}$, TPi, FPi, Haus. dist., and Compute mins. denote the simulation number, average optimal rank, true change point(s), true positive withinitime points, false positives outsideitime points, Hausdorff distance within 10 time points, and average compute time in minutes, respectively.Kin the NCPD table denotes the number of clusters used for the spectral clustering algorithm. Bolded values indicate the best performance for each metric across methods.
